# About half of older adults have two or more chronic conditions at the same time: a systematic review and meta-analysis

**DOI:** 10.3389/fpubh.2025.1680745

**Published:** 2025-12-11

**Authors:** Xianshang Zhu, Zengrui Wang, Xia Yang, Zong Ning

**Affiliations:** Department of General Medicine, The First Affiliated Hospital of Guangxi Medical University, Nanning, China

**Keywords:** multimorbidity, older adults, prevalence, risk factors, meta-analysis

## Abstract

**Background:**

Population aging has led to a rising prevalence of multimorbidity among older adults, posing a significant global public health challenge. This study aims to comprehensively integrate the evidence regarding the prevalence of multimorbidity among the older adults and the influencing factors through a systematic review and meta-analysis.

**Methods:**

We conducted a systematic review and meta-analysis in accordance with Preferred Reporting Items for Systematic Reviews and Meta-Analyses (PRISMA) guidelines. The research question was structured using the PICOS framework: **P**opulation: adults aged ≥60 or ≥65; **I**ntervention/Phenomenon of Interest: multimorbidity (≥2 chronic conditions); **C**omparator: not applicable (prevalence synthesis) or various demographic groups (risk factor analysis); **O**utcomes: prevalence of multimorbidity and pooled odds ratios (OR) of risk factors; **S**tudy design: cross-sectional studies. We conducted a comprehensive search of multiple databases up to July 2025 to identify cross-sectional studies that reported the prevalence and/or risk factors of multimorbidity (defined as ≥2 chronic conditions) in adults aged ≥60 or ≥65. The pooled prevalence was estimated using a random-effects model, and heterogeneity was explored through subgroup analysis and meta-regression.

**Results:**

Forty-nine studies involving 729,043 older adults were included. The global pooled prevalence of multimorbidity was 46.0% (95% CI: 38.0%−55.0%), with substantial heterogeneity (*I*^2^ = 99.98%). Meta-regression identified survey location as a key source of heterogeneity. Subgroup analyses revealed a higher prevalence in females (48%) than males (41%) and a lower prevalence in studies from China (40%) compared to other countries (53%). Significant risk factors included age ≥ 70 years (OR = 1.43), female sex (OR = 1.59), BMI ≥ 28 kg/m^2^ (OR = 1.97), low education (OR = 1.44), single status (OR = 1.32), and low income (OR = 1.46).

**Conclusion:**

Multimorbidity is highly prevalent among the global older adults, with significant geographical and demographic variations. Modifiable risk factors such as obesity, low education, and low income are crucial targets for intervention to alleviate the burden of multimorbidity.

## Introduction

Global population aging has become an important social challenge in the 21st century (1). According to statistics from the World Health Organization (WHO), the global proportion of people aged 60 and over has increased significantly over the past few decades, rising from 9.2% in 2000 to 12.3% in 2020; this age group is expected to account for 20% of the global population by 2050 (2). The trend of population aging is not only obvious in developed countries, such as Japan, Germany and Italy, but also gradually showing this feature in developing countries, such as China and India (3), with the latter accounting for over 60% of the global older adults population by 2030 (4).

Population aging directly increases the pressure on public health systems, as the risk of chronic diseases rises significantly with age. Multimorbidity—defined as the coexistence of two or more chronic conditions (5)—is particularly prevalent in the older adults, driven by physiological changes (e.g., immunosenescence, inflammaging), unhealthy lifestyles, and long-term accumulation of disease risk (6). A systematic review has shown that the prevalence of multimorbidity in adults aged ≥ 65 years exceeds 70% in high-income countries (7), and this coexistence of chronic diseases not only impairs patients' quality of life (e.g., reduced mobility, increased disability) but also raises medical costs—patients with multimorbidity have 2–5 times higher healthcare expenditures than those with a single chronic disease, imposing heavy burdens on individuals, families, and societies (8).

In view of the high prevalence rate of multimorbidity in the global older adults population and the serious health burden brought by it, more and more researchers have begun to pay attention to the phenomenon of multimorbidity in the older adults and conducted a large number of related studies (9). Although previous meta-analyses have documented the prevalence of multimorbidity in older adults, significant research gaps remain. First, most early reviews only reported prevalence as a broad range (e.g., 30%−70%) without calculating a pooled estimate (10), which limits cross-study quantitative comparison and fails to provide a global reference value (11). Second, many studies focused solely on prevalence and neglected quantitative analysis of risk factors—for example, only describing “age and gender are associated with multimorbidity” but not reporting pooled odds ratios (OR) or 95% confidence intervals (CI) (12). Third, there is substantial heterogeneity in the definition of multimorbidity across studies: some defined it as ≥2 chronic conditions, others as ≥3, and a few even included acute diseases, leading to inconsistent prevalence estimates that are difficult to synthesize (13). Fourth, existing reviews often have geographical or language restrictions: most focused on high-income countries in Europe and North America, and English-language publications dominated, overlooking a large body of evidence from low- and middle-income countries (e.g., China, India, Vietnam) and non-English literature, which may introduce selection bias (14). Finally, some studies restricted the age range to ≥65 years (15), excluding data on adults aged 60–64 years—a group with increasing multimorbidity risk in developing countries—resulting in incomplete population coverage. Consequently, a comprehensive, up-to-date meta-analysis that synthesizes global prevalence data from both English and Chinese literature and concurrently investigates key risk factors is urgently needed.

The primary aim of this study is to fill the above gaps by synthesizing global evidence on multimorbidity in older adults. To this end, we will conduct a systematic synthesis and meta-analysis of cross-sectional studies to determine the global prevalence of multimorbidity in older adults and its key influencing factors. Ultimately, we aim to generate robust scientific evidence to support the formulation of targeted interventions and health policies, contributing to the global response to the challenges of aging and multimorbidity.

## Materials and methods

### Registration

This study was prepared in strict accordance with the PRISMA declaration specification. To elaborate on the specific reporting content of this study, the items in the PRISMA 2020 checklist are listed in Supplementary Table 1. In addition, detailed information on the study protocol was made available online through the Prospective Register for Systematic Reviews (https://www.crd.york.ac.uk/PROSPERO/view/CRD42024602631, PROSPERO: CRD42024602631).

### Research question and PICOS framework

The research question was explicitly defined according to the PICOS framework:

Population: community-dwelling or institutionalized older adults, defined as individuals aged 60 years and over or 65 years and over.

Intervention/Phenomenon of Interest: The presence of multimorbidity, defined as the coexistence of two or more chronic non-communicable diseases.

Comparator: for the prevalence synthesis, no comparator was applicable. For the analysis of influencing factors, comparisons were made between different demographic and socioeconomic groups (e.g., male vs. female, high vs. low education).

Outcomes: the primary outcome was the prevalence of multimorbidity. Secondary outcomes were the pooled effect sizes (Odds Ratios, OR) of factors associated with multimorbidity.

Study design: observational cross-sectional studies specifically designed to assess prevalence.

### Search strategy

A comprehensive systematic search was performed in PubMed, Web of Science, Embase, Cochrane Library, CNKI, WANFANG, VIP, and CBM from their inception to July 30, 2025. The inclusion of the four major Chinese databases (CNKI, WANFANG, VIP, and CBM) was essential to capture the extensive body of relevant literature published in Chinese on multimorbidity among the older adults in China, which is a key population of interest in this global analysis and is often underrepresented in international reviews that rely solely on English-language databases (16). The search strategy was developed and optimized iteratively based on the PICO (Population, Phenomenon of Interest, Study Design) framework with the assistance of an experienced medical librarian to ensure comprehensiveness and precision. To balance sensitivity and specificity, we employed a combination of Medical Subject Headings (MeSH/Entree) and free-text terms for the key concepts: (1) Population (P): terms related to older adults (e.g., “Aged”, “Elderly”, “Older adults”, “Older people”, “Geriatric”); (2) phenomenon of Interest (*I*): terms related to multimorbidity (e.g., “Multimorbidity”, “Comorbidity”, “Multiple chronic conditions”, “Multiple long-term conditions”, “Multiple morbidity”); (3) study design (*O*): terms related to study design and outcome (e.g., “Cross-Sectional Studies”, “Prevalence”, “Epidemiology”). Within each conceptual group, synonyms and related terms were combined using the Boolean operator “OR”. The three conceptual groups were then combined using the Boolean operator “AND” to form the final search strategy. The detailed search strategies for each database are provided in Supplementary Table 2. Furthermore, to ensure comprehensiveness, we supplemented the database searches with a literature tracking approach by manually reviewing the reference lists of all included articles to identify any additional studies that met our eligibility criteria.

### Inclusion and exclusion criteria

Studies were included in this meta-analysis if they met all of the following criteria: (1) Population: the study population consisted of older adults, defined as individuals aged 60 years and over or 65 years and over. (2) Concept: this study specifically examined the phenomenon of “coexistence of multiple diseases”, which refers to the situation where an individual suffers from two or more chronic non-communicable diseases simultaneously (such as hypertension, diabetes, COPD, etc.). (3) Study design: the study employed a cross-sectional design (To ensure the comparability and quality of prevalence data, this meta-analysis exclusively included cross-sectional studies that were specifically designed to assess prevalence. While baseline data from longitudinal studies were considered, they were ultimately excluded because their primary objective is typically not to report prevalence, which can lead to inconsistent data availability and potential bias in baseline reporting). (4) Outcomes: the study reported at least two of the following outcomes: the prevalence of multimorbidity, or provided sufficient data for its calculation. Risk factors for multimorbidity, assessed using logistic regression and reported as Odds Ratios (OR) with corresponding 95% Confidence Intervals (CI). Studies were excluded based on the following criteria: (1) Unclear definitions or data: studies with an ambiguous definition of multimorbidity or unclear descriptions of the specific chronic conditions assessed. (2) Inappropriate study design: studies that were not cross-sectional (e.g., cohort studies, case-control studies, randomized controlled trials). (3) Publication type: non-original research articles, such as case reports, reviews, editorials, commentaries, conference abstracts, or letters to the editor. (4) Language: articles published in languages other than English or Chinese. (5) Data accessibility: studies for which the full text was unavailable, or from which essential data could not be extracted or calculated. To prevent data duplication from overlapping populations, if multiple studies were identified from the same region or cohort, we included only the study with the largest sample size or the most recently published data.

### Study selection and data extraction

All literature in the databases was imported into Endnote software for literature selection and management. First, duplicate publications were removed. Subsequently, two researchers independently conducted a preliminary screening of the titles and abstracts according to the set inclusion and exclusion criteria to exclude irrelevant references. Finally, the researchers read the full text and screened again to determine the final literature to be included. If differences of opinion arose between the two researchers during this process, these differences were resolved through negotiation or consultation with a third researcher. After determining the selected research list, two independent researchers conducted two rounds of data extraction for each included study to minimize errors. Data extraction was carried out using a standardized Microsoft Excel spreadsheet, which included: first author, publication year, survey time, survey location, study design, sample size, sample gender ratio, prevalence of chronic diseases, prevalence of multimorbidity, and relevant influencing factors. The relevant influencing factors include: age, gender, marital status, body mass index, educational level, smoking status, drinking status, exercise habits, income level, and place of residence. For studies that reported the number of individuals with two or more chronic conditions but did not explicitly report the prevalence percentage, the prevalence was calculated during data extraction using the formula: prevalence (%) = (number of individuals with multimorbidity/total study sample size) × 100%. This calculated value was then used in the meta-analysis.

### Methodological quality assessment

Two researchers independently assessed the methodological quality of the final included literature. They systematically scored the included studies using the cross-sectional study evaluation criteria recommended by the Agency for Healthcare Research and Quality (AHRQ) (17). The AHRQ methodological checklist is widely recognized as an effective tool for assessing the quality of cross-sectional studies and is currently accepted and used by researchers in multiple fields (18). The checklist can be found at http://www.ncbi.nlm.nih.gov/books/NBK35156/. The tool contains 11 assessment items relating to data sources, inclusion and exclusion criteria, timing and order of inclusion of participants, influence of evaluator subjective factors, assessment of quality assurance, interpretation of data exclusions, control for confounding variables, management of missing data, integrity of data collection, and follow-up. Each assessment item is graded with “Yes”, “No” or “Unclear”, where “Yes” was awarded 1 point, and the remaining options were not awarded points. Depending on the score, a score of 0–3 indicated “low” methodological quality, a score of 4–7 indicated “medium” methodological quality, and a score of 8–11 indicates “high” methodological quality. In addition, the process of quality evaluation was independently conducted by two researchers, and the consistency of their scores was evaluated by Cohen's Kappa coefficient (19). For literature with inconsistent evaluation results, the researchers resolved these differences by discussing them with a third investigator.

### Subgroup analysis and meta-regression

To investigate potential sources of the significant heterogeneity observed among the included studies, we performed both subgroup analyses and meta-regression. These analyses were pre-specified to explore the influence of key study-level characteristics on the pooled prevalence of multimorbidity. The moderators examined included age, gender, study quality (high vs. medium), survey region, survey year, and sample size. We aimed to assess whether these factors significantly influenced the overall pooled estimate.

### Data analysis and synthesis

In this study, Stata 18.0 software was used to conduct a meta-analysis of the included literatures. To ensure the consistency of the analysis, all the included studies met the “multimorbidity definition” criteria. We extracted the prevalence of multimorbidity among the older adults from the included studies. When the prevalence of multimorbidity was not explicitly reported, it was calculated using the formula: prevalence = (number of individuals with ≥ 2 chronic conditions)/(total number of participants) × 100%, following the method used in previous meta-analyses (20). Pooled results were estimated using a random-effects model for the prevalence of multimorbidity and reported using weighted point estimates based on the prevalence of multimorbidity and 95% confidence intervals (95% CI). The heterogeneity among the included studies was assessed using the Chi-squared (χ^2^) test, with a significance level of *P* < 0.10. The magnitude of heterogeneity was quantified by the *I*^2^ statistic. If *I*^2^ ≤ 50% and *P* > 0.10, the heterogeneity among studies was considered small, and the fixed-effect model was adopted. If *I*^2^ > 50% and *P* ≤ 0.10, it indicated moderate or high heterogeneity among studies, and a random-effects model was used (21). We performed a sensitivity analysis to test the robustness of the results by excluding one study at a time and observing changes in the pooled prevalence in the remaining studies. For the pooled effect size of the influencing factors, the combined OR value and 95% CI of the influencing factors were obtained by comparing the fixed-effect model and the random-effect model. If the results of the two models are similar, it can be inferred that the results of this study have good reliability and stability. In addition, the funnel plot and Egger's test were used to evaluate potential publication bias. A result of *P* > 0.05 was considered to indicate a low risk of publication bias, while *P* < 0.05 indicated statistically significant asymmetry.

## Results

### Search results

**Figure 1:**
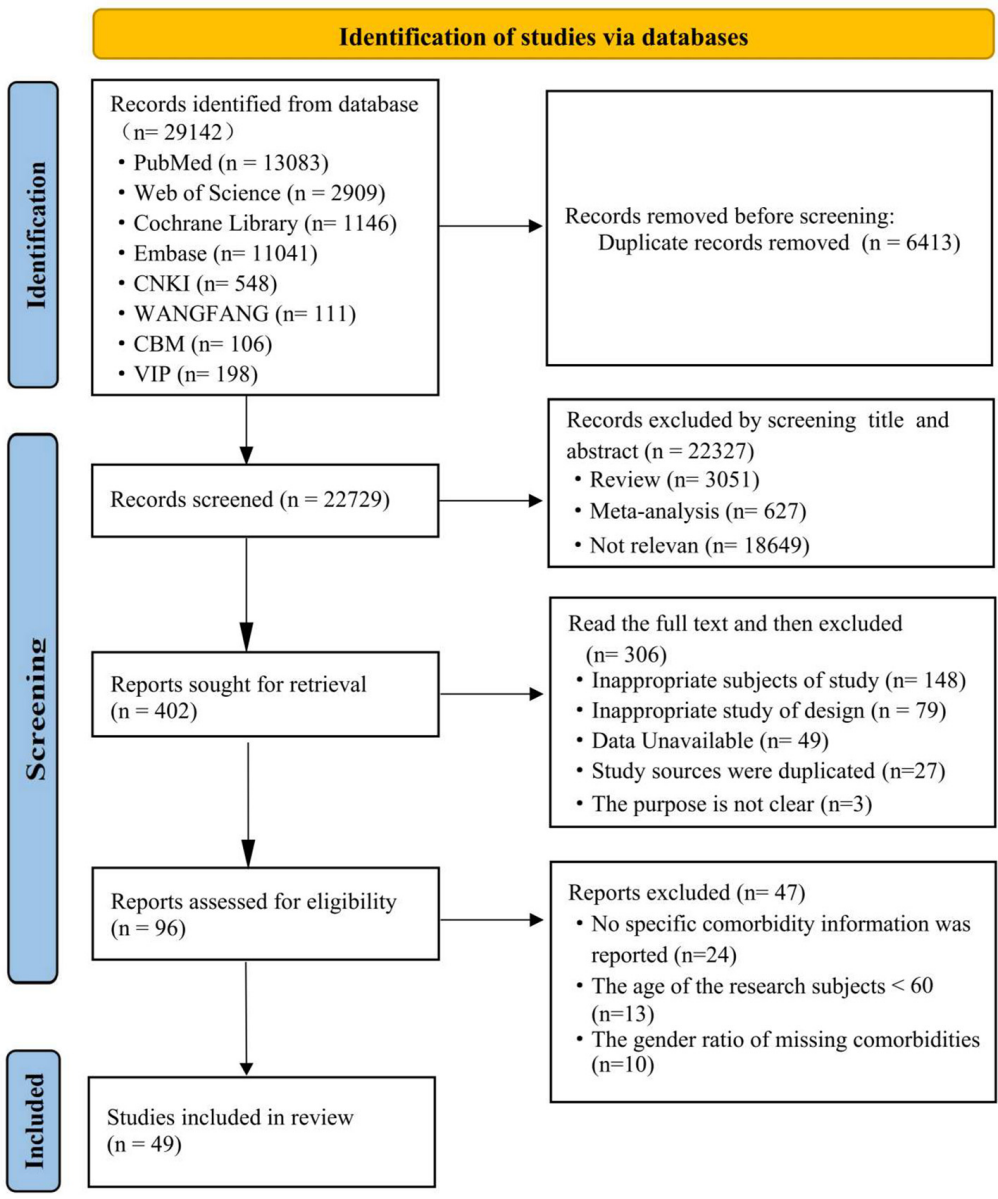
PRISMA flow diagram of literature search and selection.

The literature screening and selection process is illustrated in the PRISMA flow diagram (Figure 1). Our initial search across all databases yielded a total of 29,142 records. After removing 6,413 duplicates, 22,729 unique articles remained for screening.

In the first stage of screening, we reviewed titles and abstracts, which led to the exclusion of 18,649 records irrelevant to the research topic and 3,678 records of an inappropriate study type (e.g., reviews, case reports, editorials). This process left 402 articles for full-text eligibility assessment.

Upon detailed full-text review, a further 306 articles were excluded for the following reasons: non-representative sample or setting (*n* = 148), inappropriate study design (*n* = 79), data unavailable (*n* = 49), use of previously published data (*n* = 27), and unclear research objectives (*n* = 3).

This left 96 articles for the final eligibility check. From this pool, we excluded 34 studies due to missing essential data, such as specific comorbidity information or sex-specific prevalence, and an additional 13 studies because their study population included participants younger than 60 years of age.

Ultimately, 49 original studies with complete data on the prevalence and/or risk factors of multimorbidity in older adults were included in the final meta-analysis.

### Studies characteristics

All features included in this study are shown in Table 1. Finally, 49 studies were selected for analysis. The studies included 729,043 older adults respondents, of whom 347,779 had two or more chronic conditions at the same time. The studies were published between 2010 and 2022, with sample sizes ranging from 279 to 229,493 people. Of the 49 studies, 26 were from China, while the remaining 23 were from 21 countries, including the United States, Japan, South Korea, India, Vietnam, Germany, Switzerland and others. Of these, 17 studies clearly reported various factors influencing multimorbidity. All included studies scored from a minimum of 4 to a maximum of 8 on the quality assessment.

**Table 1:** Characteristic summary of included studies on the prevalence of multimorbidity in the older adults.

**Author, year**	**Area**	**Time of survey**	**Sampling method**	**Sample size, (*n*)**	**Age**	**Gender**		**Quality score**	**Comorbidity rate, *n* (%)**	**Influencing factors of comorbidity**	**References**
						**Male**, ***n*** **(%)**	**Female**, ***n*** **(%)**				
Liu YT et al. 2022	China	2019	Cluster sampling	1,552	≥60	837 (53.92)	715 (46.08)	8	657 (42.33)	1, 5	(22)
Chen YT et al. 2023	China	2018	Convenience sampling	1,262	≥60	613 (48.57)	649 (51.43)	8	275 (21.79)	3, 4, 8, 9	(36)
Li GX et al. 2023	China	2021	Random sampling	1,310	≥60	586 (44.73)	724 (55.27)	8	685 (52.29)	7	(52)
Guo D et al. 2022	China	2018	Stratified quota sampling	7,507	≥65	3,734 (49.74)	3,773 (50.26)	7	5,383 (71.71)	1, 3	(37)
Kong Y et al. 2021	China	2021	Multistage cluster sampling	10,062	≥60	4,930 (48.99)	5,132 (51.01)	4	2,007 (19.95)	/	(50)
Li X et al. 2019	China	2017	Multi-stage stratified random sampling	4,833	≥60	2,199 (45.49)	2,634 (54.51)	6	778 (16.10)	/	(51)
Zhang H et al. 2019	China	2015	Multistage stratified cluster sampling	23,718	≥60	10,533 (44.41)	13,185 (55.59)	5	13,097 (55.22)	/	(56)
Qi YT et al. 2023	China	2018	Multilevel random sampling	7,354	≥60	3,727 (50.68)	3,627 (49.32)	7	4,792 (65.16)	1, 2, 5, 9, 10	(23)
Liu XX et al. 2023	China	2019	Multi-stage random sampling	107,177	≥60	48,841 (45.57)	58,336 (54.43)	5	14,511 (13.54)	/	(53)
Tian L et al. 2023	China	2021	Cluster sampling	15,899	≥65	6,787 (42.69)	9,112 (57.31)	5	5,476 (34.44)	/	(54)
Zhu PY et al. 2023	China	2019	Multi-stage random sampling	8,335	≥60	3,484 (41.79)	4,851 (58.21)	7	1,385 (16.62)	1, 3, 5–9	(24)
Yu ZJ et al. 2023	China	2020	/	1,849	≥60	801 (43.32)	1,048 (56.68)	4	1,021 (55.22)	1, 3	(59)
He YZ et al. 2023	China	2021	Cluster sampling	2,010	≥65	963 (47.91)	1,047 (52.09)	8	623 (30.99)	1, 2, 4, 9	(84)
Wang WH et al. 2024	China	2021	Multistage stratified cluster sampling	20,724	≥65	9,787 (47.22)	10,937 (52.78)	5	9,759 (47.09)	/	(61)
Mu YJ et al. 2023	China	2019	Cluster sampling	4,475	≥65	1,905 (42.56)	2,570 (57.44)	8	2,728 (60.96)	/	(60)
Yao YL et al. 2022	China	2020	/	2,506	≥60	1,041 (41.54)	1,465 (58.46)	4	566 (22.59)	/	(62)
Cao M et al. 2021	China	2019	Multistage stratified cluster sampling	1,336	≥60	645 (48.28)	691 (51.72)	8	490 (36.68)	/	(57)
Hou YT et al. 2020	China	2016	Multi-stage random sampling	622	≥65	264 (42.44)	358 (57.56)	6	317 (50.97)	1–3, 4, 9	(85)
Liu DN et al. 2023	China	2017	Multi-stage stratified random sampling	12,507	≥60	5,298 (48.25)	7,209 (51.75)	5	6,925 (55.37)	/	(58)
Zhang XQ et al. 2024	China	2021	/	1,491	≥60	596 (39.97)	895 (60.03)	8	1,192 (79.95)	/	(55)
Zhou FK et al. 2023	China	2018–2020	Cluster sampling	8,221	≥65	3,772 (45.88)	4,449 (54.12)	6	2,402 (29.22)	/	(63)
Marzban M et al. 2024	Iran	2015	/	2,426	≥60	1,161(47.85)	1,265(52.15)	5	1,946 (80.21)	2, 4, 5, 7, 8, 9	(86)
Oliveira-Figueiredo DST et al. 2024	Brazil	2020	Random sampling	22,728	≥60	10,193 (44.84)	12,535 (55.16)	7	11,728 (51.60)	1–3, 5, 6	(25)
Ko S et al. 2024	India	2018	Multi-stage stratified sampling	12,316	≥60	6,158 (50.00)	6,158 (50.00)	8	3,062 (24.86)	/	(64)
Su W et al. 2024	China	2020–2021	Stratified random sampling	1,161	≥60	499 (42.98)	662 (57.02)	8	366 (31.53)	1, 8, 9	(83)
Kohler S et al. 2024	Dares Salaam	2017–2018	Random sampling	555	≥60	243 (43.78)	312 (56.21)	7	465 (83.78)	/	(65)
Lee C et al. 2024	Korea	2019	/	1,041	≥65	233 (22.38)	808 (77.62)	6	936 (89.91)	/	(66)
Maimaitiwusiman Z et al. 2023	China	2019	Multi-stage random sampling	86,510	≥60	41,188 (47.61)	45,322 (52.39)	7	28,894 (33.40)	/	(67)
Reyes-Ortiz CA et al. 2023	Colombia	2015	Multi-stage stratified random sampling	18,873	≥60	8,757 (46.39)	10,116 (53.61)	7	8,172 (43.30)	1–3, 5, 7, 10	(26)
You L et al. 2023	China	2022	Multistage stratified cluster sampling	7,774	≥60	3,876 (49.89)	3,898 (50.11)	8	3,830 (49.27)	/	(68)
Yang K et al. 2023	China	2021	Convenience sampling	4,803	≥60	2,276 (47.38)	2,527 (52.62)	8	1,883 (39.21)	/	(69)
Honda Y et al. 2022	Japan	2013	Stratified random sampling	23,340	≥65	10,266 (43.98)	13,074 (56.02)	5	9,574 (41.02)	/	(70)
Lynch DH et al. 2022	United States	2005–2014	Multi-stage random sampling	7,261	≥60	3,652 (50.30)	3,609 (49.70)	6	4,965 (68.38)	/	(71)
Balakrishnan S et al. 2022	Eastern Nepal	2020	Multistage cluster sampling	843	≥60	431 (51.12)	412 (48.88)	8	192 (22.78)	1, 5, 6–8, 10	(27)
Keomma K et al. 2022	São Paulo	2015	Complex probability sampling	1,019	≥60	387 (37.97)	632 (62.03)	7	408 (40.04)	2, 5, 9	(28)
Shariff Ghazali S et al. 2021	Malaysia	2018	Two-stage stratified cluster sampling	3,966	≥60	1,868 (47.10)	2,098 (52.90)	5	1,543 (38.91)	/	(72)
Lin WQ et al. 2022	China	2020	Multi-stage stratified random sampling	31,708	≥65	14,046(44.29)	17,662 (55.71)	5	4,819 (15.20)	/	(73)
Sara HH et al. 2018	Bangladesh	2017	Two-stage stratified random sampling	566	≥60	432 (76.32)	134 (23.68)	7	319 (56.36)	/	(74)
Smith L et al. 2022	Irish	2009–2011	Multistage stratified cluster sampling	2,941	≥65	1,323 (44.98)	1,618 (55.02)	8	2,041 (69.40)	/	(75)
Jovic D et al. 2016	Serbia	2013	Two-stage stratified random sampling	2,749	≥65	1,174 (42.70)	1,575 (57.30)	5	1,578 (57.40)	/	(76)
Puth MT et al. 2017	Germany	2012–2013	Two-stage random sampling	7,152	≥60	3,454 (48.29)	3,698 (51.71)	5	4,882 (68.26)	/	(77)
Bähler C et al. 2015	Switzerland	2013	/	229,493	≥65	98,124 (42.76)	131,369(57.24)	5	175752 (76.58)	/	(78)
Ha NT et al. 2015	Vietnam	2010	Multi-stage random sampling	2,400	≥60	834 (34.75)	1,566 (65.25)	7	941 (39.21)	1–6, 8, 10	(29)
Yadav UN et al. 2021	Nepal	2018	Multistage cluster sampling	794	≥60	400 (50.38)	394 (49.62)	8	116 (14.61)	/	(79)
Hien H et al. 2014	Burkina Faso	2012	Random sampling	389	≥60	215 (55.27)	174 (44.73)	8	252 (64.78)	/	(80)
Aye SKK et al. 2019	Myanmar	2016	Multi-stage random sampling	4,859	≥60	1,841 (37.89)	3,018 (62.11)	7	1,613 (33.20)	2, 4, 5, 6	(87)
Asante D et al. 2022	Australia	2013–2017	Random sampling	5,920	≥60	2,442 (41.25)	3,478 (58.75)	7	1,973 (33.33)	/	(81)
Abdulazeez ZU et al. 2021	Nigeria	2018	Random sampling	279	≥60	93 (33.40)	186 (66.70)	8	201 (72.04)	/	(82)
Nugraha S et al. 2020	Indonesia	2018	Random sampling	427	≥60	137 (32.08)	290 (67.92)	5	259 (60.66)	/	(83)

1: senior age, 2: gender (female), 3: obesity, 4: educational level (high), 5: smoking, 6: drinking, 7: exercise situation, 8: marital status, 9: income level, 10: place of residence.

### Methodological quality assessment results

The quality evaluation results of this study showed that 16 studies (32.65%) were rated as high quality, while 33 studies (67.35%) were rated as medium quality. All included studies defined multimorbidity and were cross-sectional studies. More than half of the studies (51.02%) clarified the inclusion and exclusion criteria for participants. In most of the included studies, assessments to assure study quality (89.79%) and summaries of patient response rates and data integrity (71.43%) were reported. However, none of the studies described the impact of the evaluators' subjective factors on the findings, nor did they detail how missing data was dealt with in their analyses. Details are given in supplementary Table 3.

### Meta-analysis of the prevalence of multimorbidity

**Figure 2:**
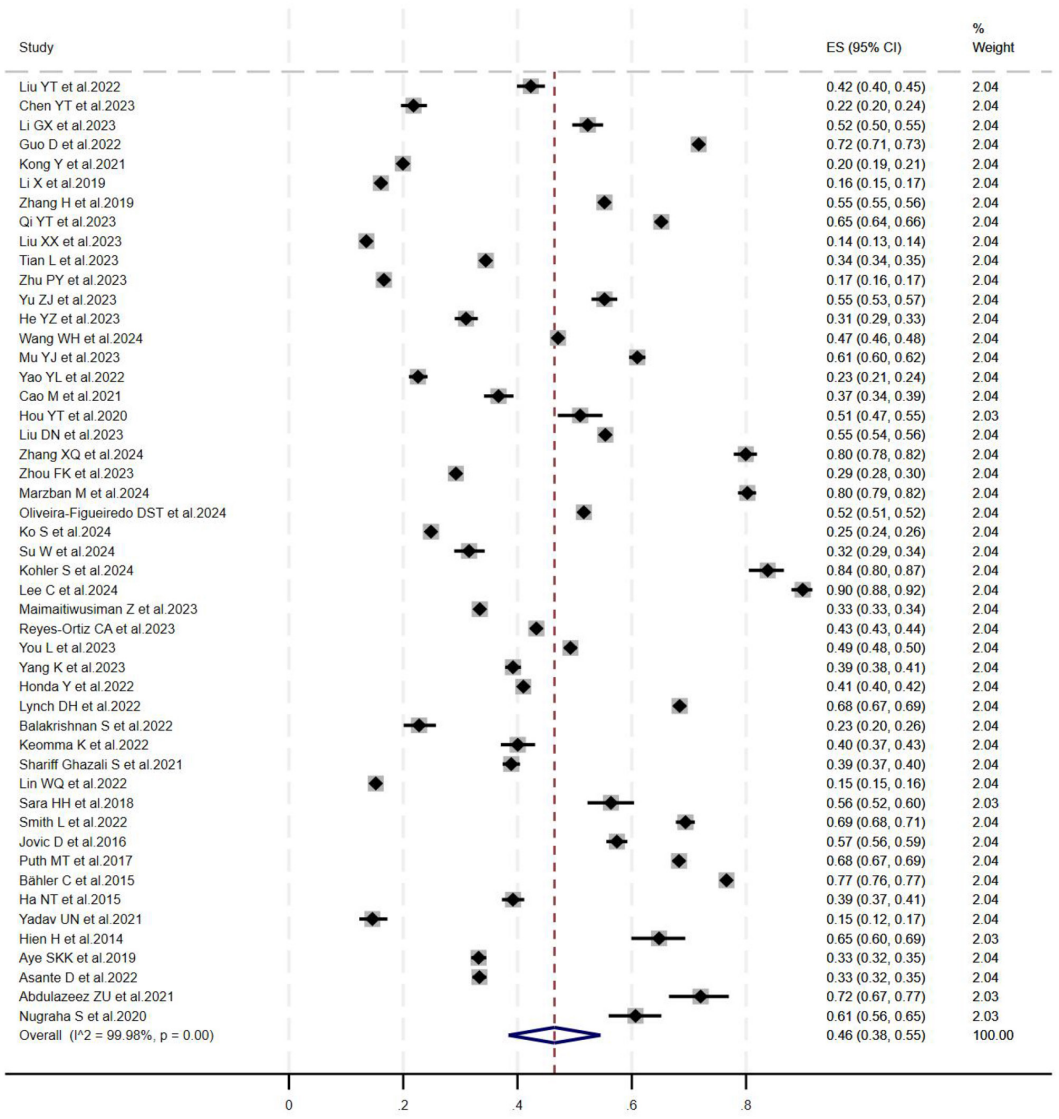
Meta-analysis of the prevalence of multimorbidity in the older adults.

A meta-analysis of the prevalence of multiple comorbidities was conducted on the 49 included studies using a random-effects model. The results showed that the overall combined prevalence based on all 49 studies (*n* = 729,043) was 46.0% (95% CI: 38.0%−55.0%), with extremely high heterogeneity (*I*^2^ = 99.98%, *P* = 0.001). The forest map is shown in Figure 2.

To explore the sources of heterogeneity, we performed additional analyses to examine the prevalence of multimorbidity using subgroup analysis and meta-regression methods. The results of our subgroup analysis indicated that gender, age, research quality, study location, survey time, and sample size were not important factors affecting the heterogeneity of this meta-analysis. Although most subgroup differences did not reach statistical significance, we retained Table 2 to transparently present the direction and magnitude of heterogeneity across study characteristics. These findings help identify potential sources of clinical and methodological diversity, which are informative for future research design and policy planning. However, meta-regression results showed that study location was an important factor affecting the heterogeneity of this meta-analysis.

**Table 2:** Subgroup analysis and Meta-regression analysis of the prevalence of multimorbidity in the older adults.

**Analysis**	**Studies, *n***	**Subgroup**				**Meta-regression**		
		**Pooled estimate**	**95% CI**	*I* ^2^	***P*** **-value**	**Coefficient**	**95% CI**	***P*** **-value**
Gender						0.072	(−0.018, 0.161)	0.117
Male	37	0.41	(0.35–0.47)	99.88%	< 0.001			
Female	37	0.48	(0.41–0.55)	99.93%	< 0.001			
Age						0.074	(−0.059, 0.208)	0.270
≥60	36	0.44	(0.38–0.51)	99.95%	< 0.001			
≥65	13	0.52	(0.35–0.69)	99.99%	< 0.001			
Research quality						−0.071	(−0.219, 0.076)	0.334
Medium	33	0.48	(0.39–0.57)	99.99%	< 0.001			
High	16	0.41	(0.27–0.54)	99.88%	< 0.001			
Study location						0.132	(0.019, 0.246)	0.023
China	26	0.40	(0.33–0.47)	99.96%	< 0.001			
Other	23	0.53	(0.44–0.63)	99.95%	< 0.001			
Survey time						−0.098	(−0.227, 0.031)	0.133
~2020	35	0.49	(0.39–0.59)	99.99%	< 0.001			
2021~	14	0.39	(0.30–0.49)	99.92%	< 0.001			
Sample size						0.138	(−0.047, 0.324)	0.141
10,000~	13	0.39	(0.23–0.56)	100.00%	< 0.001			
1,001–9,999	28	0.48	(0.40–0.56)	99.89%	< 0.001			
~1,000	8	0.53	(0.38–0.55)	99.59%	< 0.001			

### Sensitivity analysis

The robustness of the findings was assessed by the leave-one method, and each study was individually excluded for analysis. The prevalence of multimorbidity in the older adults ranged from 45.56% to 47.15%, indicating that the overall prevalence of comorbidity fluctuated less, which meant that the study results were stable and reliable. In addition, a sensitivity analysis of 33 medium-quality studies showed that the prevalence of multimorbidity in the older adults ranged from 46.82% to 48.83%, further suggesting that the findings were also stable and reliable in medium-quality studies.

### Publication bias

**Figure 3:**
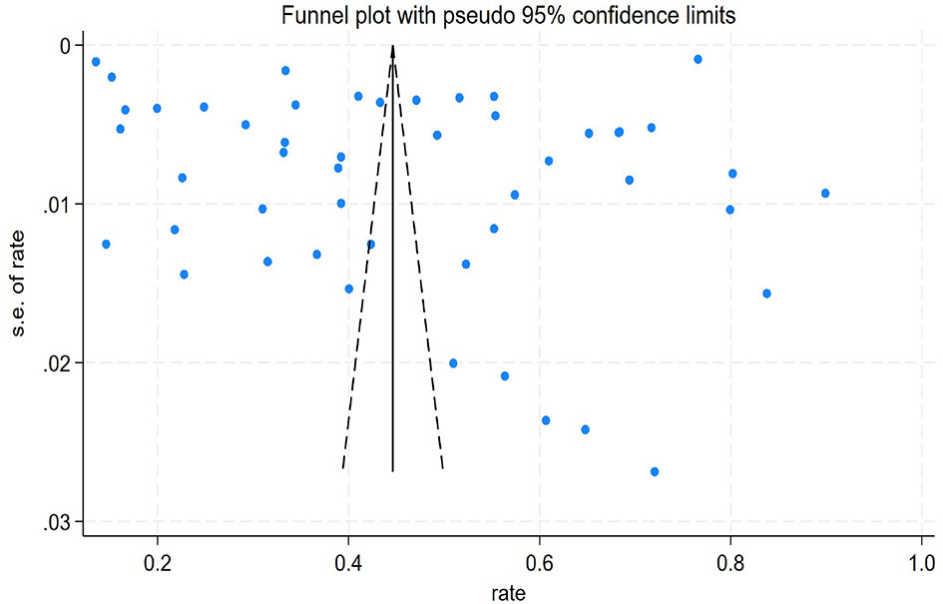
Funnel plot of publication bias.

Potential publication bias was assessed by visual inspection of a funnel plot and formally tested using Egger's linear regression test. The funnel plot, which plots the study effect size against its standard error, appeared to be largely symmetrical around the pooled effect estimate (Figure 3). However, given the substantial heterogeneity present in this meta-analysis, the interpretation of the funnel plot's symmetry should be approached with caution, as heterogeneity can be a confounding factor for plot asymmetry. To supplement the visual assessment, Egger's test was performed. The result did not provide statistical evidence of significant publication bias (*P* = 0.798). While this statistical test supports the visual inspection, the potential influence of heterogeneity on these assessments cannot be entirely ruled out.

### Meta-analysis of influencing factors of multimorbidity

Of the 49 studies included, 17 examined factors influencing multimorbidity in older adults. Through the extraction of data from these 17 studies, 10 risk factors associated with multimorbidity were identified and their effect sizes were pooled for analysis. The results of the analysis showed that the following factors were identified as significant risk factors for multimorbidity in older adults: age (≥70 years), gender (female), body mass index (BMI ≥ 28kg/m^2^), education level (at least junior high school level), marital status (single, widowed, divorced), and higher income level. However, this study did not find that smoking, alcohol consumption, lack of exercise, and place of residence had significant effects on the occurrence of multimorbidity. Detailed data are shown in Table 3.

**Table 3:** Results of meta-analysis of risk factors for multimorbidity in the older adults.

**Risk factors**	**Studies, *n***	**Heterogeneity**		**Effect model**	**Pooled estimate**			
		***P***	*I*^2^ **(%)**		**OR**	**95% CI**	***Z***	***P***
Age (≥70)	11	0.001	96.46	random	1.43	1.09–1.89	2.57	0.010^*^
Gender (Female)	9	0.001	86.80	random	1.59	1.38–1.83	6.44	0.001^*^
BMI (≥28.0 kg/m^2^)	7	0.001	96.73	random	1.97	1.28–3.03	3.10	0.002^*^
Educational	6	0.072	55.18	random	0.56	0.43–0.73	−4.36	0.001^*^
Smoking	10	0.006	62.92	random	1.09	0.99–1.20	1.71	0.088
Tipple	5	0.001	82.80	random	0.88	0.64–1.20	−0.81	0.416
Exercise (lack)	5	0.001	95.46	random	0.82	0.52–1.30	−0.85	0.397
Marital status	6	0.004	80.44	random	1.32	1.02–1.71	2.09	0.037^*^
Income (low)	8	0.001	83.93	random	1.46	1.11–1.91	2.71	0.007^*^
Live in the city	4	0.001	96.35	random	1.09	0.64–1.83	0.31	0.757

CI, confidence interval; ^*^P < 0.05

### Sensitivity analysis

In this study, sensitivity analysis was conducted under fixed effects model and random effects model for OR values and 95%CI of each influencing factor. The results showed that although there were some differences in the calculation results of smoking and exercise, the OR values and 95%CI of the two models showed significant agreement in the analysis of other influencing factors. This finding indicates that the meta-analysis results of influencing factors in this study have good stability, and the details are shown in Table 4.

**Table 4:** Sensitivity analysis of risk factors.

**Risk factors**	**Fixed effect model**			**Random effects model**		
	**OR**	**95% CI**	***P***	**OR**	**95% CI**	***P***
Age (≥70)	1.21	1.17–1.26	0.001^*^	1.43	1.09–1.89	0.010^*^
Gender (Female)	1.43	1.38–1.49	0.001^*^	1.59	1.38–1.83	0.001^*^
BMI (≥28.0kg/m^2^)	1.57	1.47–1.68	0.001^*^	1.97	1.28–3.03	0.002^*^
Educational	0.58	0.51–0.67	0.001^*^	0.56	0.43–0.73	0.001^*^
Smoking	1.13	1.09–1.17	0.001^*^	1.09	0.99–1.20	0.088
Tipple	0.92	0.82–1.03	0.155	0.88	0.64–1.20	0.416
Exercise (lack)	1.12	1.03–1.22	0.011^*^	0.82	0.52–1.30	0.397
Marital status	1.21	1.09–1.34	0.001^*^	1.32	1.02–1.71	0.037^*^
Income (low)	1.51	1.37–1.66	0.001^*^	1.46	1.11–1.91	0.007^*^
Live in the city	1.05	0.96–1.15	0.257	1.09	0.64–1.83	0.757

CI, confidence interval; ^*^P < 0.05

### Publication bias

**Figure 4:**
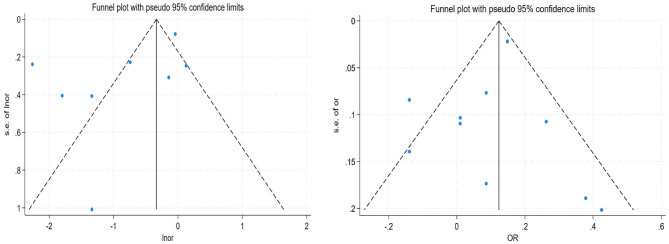
Age and smoking publication bias funnel plot, the picture on the left is age and the picture on the right is smoking.

For the overall prevalence analysis, we visually inspected the funnel plot and conducted Egger's test to assess publication bias. The funnel plot showed a roughly symmetrical distribution (Figure 3), and Egger's test did not indicate significant publication bias (*P* = 0.798). Additionally, for the “risk factors” meta-analysis, we only conducted a publication bias assessment for the factors with a large number of included studies (≥10 studies), such as age and smoking. Figure 4 shows the funnel plots for age and smoking, and their asymmetry suggests the possibility of some publication bias, which should be taken into account when interpreting the combined results of these two specific risk factors.

## Discussion

This study was the first to systematically integrate 49 Chinese and English papers from 22 countries around the world. The aim was to comprehensively understand the prevalence of multimorbidity among the older adults and the related influencing factors through meta-analysis. We found that the problem of multimorbidity is extremely common among the older adults population. We calculated that the global prevalence of multimorbidity was 46.0%, but we also found that this prevalence showed significant differences globally (*I*^2^ = 99.98%). This also indicates that using a single numerical value to represent the multimorbidity status of the global older adults population is inappropriate, and it is crucial to identify the factors causing these differences. Meanwhile, there are several factors affecting the prevalence of multimorbidity in the older adults, including age (≥70 years), gender (female), body mass index (BMI ≥ 28kg/m^2^), education level (junior high school or above), marital status (single, widowed, divorced) and higher income level. These factors have a significant impact on multimorbidity among older adults.

### Exploration of the sources of heterogeneity

Our analysis revealed that the study location was the most important factor explaining the high heterogeneity of the prevalence of multiple comorbidities. The most significant finding was that the combined prevalence rate of older adults people in China was significantly lower than the aggregated results of other countries. We suggest that this significant difference may stem from complex interactions among factors such as disease spectrum, adopted diagnostic criteria, common lifestyles, and national medical system characteristics. This study effectively overcame language barriers by systematically searching and including both Chinese and English literature, especially a large number of Chinese studies. It compensated for the shortcomings of previous reviews that were limited to English publications and might have overlooked key evidence, providing a more comprehensive and representative picture of the global prevalence of comorbidities. Furthermore, there are significant differences in the definition of multimorbidity included in the studies: some studies define it as ≥2 chronic diseases, while others adopt the standard of ≥3 diseases. This directly leads to the incomparability of prevalence estimates and is another important reason for the heterogeneity. Such inconsistencies in definition are widespread in international literature, reflecting the lack of a unified standard in this field. Future research needs to reach a consensus on this to improve the comparability of results.

### Influence of age on multimorbidity

Our meta-analysis reaffirms that advanced age is arguably the most potent, non-modifiable risk factor for multimorbidity, a finding consistent with a vast body of literature (30). The significantly increased risk observed in individuals aged 70 and over (22–26, 50–52) is deeply rooted in the fundamental biological processes of aging. This study confirmed that being 70 years old or older is a risk factor for multimorbidity (OR = 1.43, 95% CI: 1.09–1.89). This further supports the mechanism that 'aging-related physiological changes (such as cellular aging, inflammatory aging) are the core driving factors for multimorbidity (31). However, it should be noted that Smith et al. (2022) conducted a longitudinal study on older adults people in American communities (*n* = 18,721), finding that for individuals aged 60–69 who had a “hypertension + diabetes” background, the risk of multimorbidity after the age of 70 was 2.3 times higher than that of those without such a background (HR = 3.30, 95% CI: 2.91–3.75) (32). This suggests that the interaction effect of “age + cumulative underlying diseases” might have been overlooked by the cross-sectional design of this study. Future research needs to verify this interaction through longitudinal data.

### Influence of gender on multimorbidity

The study results further revealed the gender differences in the prevalence of multimorbidity in the older adults population, showing that the prevalence of multimorbidity in older adults women was significantly higher than that in older adults men (23, 25, 26, 28, 29, 50–53). The higher co-morbidity risk in women is closely related to the physiological changes after menopause and their medical-seeking behaviors. The decline in estrogen levels significantly increases women's susceptibility to various chronic diseases such as cardiovascular diseases and osteoporosis (33). In addition, women usually tend to report symptoms and seek medical help (34, 35), which may partly contribute to their higher prevalence being recorded. These two factors jointly explain the observed gender differences.

### Influence of body mass index on multimorbidity

Body mass index (BMI) is one of the important factors affecting the occurrence of multimorbidity in the older adults. Our findings suggest that the risk of multimorbidity in older adults is significantly increased when BMI exceeds 28kg/m^2^ (24–26, 29, 36, 37, 51). Obesity is the core metabolic driving factor of multiple comorbidities, mainly exerting its effects through two pathways: inflammation and insulin resistance. The pro-inflammatory cytokines secreted by adipose tissue (such as TNF-α and IL-6) establish a chronic low-level inflammatory state (38). Meanwhile, obesity directly leads to insulin resistance (39). These two systemic obstacles jointly create an internal environment that is highly prone to triggering a variety of chronic diseases (such as diabetes and cardiovascular diseases), rather than merely increasing the risk of a single disease.

### Influence of educational level on multimorbidity

Educational level influences the risk of comorbidity by enhancing health literacy and the ability to access resources. Higher educational levels not only directly empower individuals to adopt healthy behaviors (such as quitting smoking, maintaining a healthy diet) but also effectively utilize medical information (29, 36, 51, 52). It is also usually associated with a higher social and economic status, thereby ensuring better accessibility to medical resources and health protection (40, 41). These advantages collectively facilitate the early management and intervention of chronic diseases and are the key social determinants for reducing the risk of comorbidities.

### Influence of marital status on multimorbidity

Marital status significantly influences the risk of multiple diseases through social support mechanisms. Our analysis has confirmed that being unmarried (single, widowed, or divorced) is an important risk factor for multiple diseases (24, 27, 29, 36, 42, 52). Married individuals usually can obtain more stable emotional support and health supervision from their spouses, which is beneficial for the management of chronic diseases (43). Conversely, the lack of social support may exacerbate the emotional stress of non-married older adults individuals and weaken their compliance with healthy behaviors, thereby promoting the occurrence and development of multiple diseases (44). Therefore, when formulating intervention strategies, it is of utmost importance to establish a strong social support network for older adults people living alone.

### Influence of economic base on multimorbidity

Our pooled results suggest that lower income levels are an important risk factor against multimorbidity (23, 24, 28, 36, 42). Individuals with higher incomes generally have access to better quality health care services, healthier living environments, and more adequate resources to manage and manage chronic diseases (45). They were also more likely to engage in healthy lifestyles, such as regular exercise, maintaining a nutritionally balanced diet, and limiting tobacco and alcohol consumption. To address the challenges posed by economic disparities, implementing policies that reduce poverty, provide affordable housing and equitable access to health care could have a positive impact on reducing the burden of multimorbidity among older adults.

### Influence of other factors on multimorbidity

Regarding factors such as smoking and drinking, this study did not find a significant correlation between these factors and multiple diseases. However, this result should be interpreted with caution (46), this is likely to reflect the fact that the existing related studies are few in number and highly heterogeneous, resulting in insufficient statistical test power and failing to confirm that these factors are truly ‘harmless'. Therefore, we should not dismiss the importance of a healthy lifestyle based on this. Adhering to quitting smoking, limiting alcohol consumption, and maintaining regular exercise remains a fundamental strategy for the older adults to maintain their health and manage chronic diseases. Given the high prevalence of multimorbidity among the older adults, we believe that an integrated care model is necessary to address the complex health care needs of this group (47). In addition, healthcare providers should receive systematic training on the principles of geriatric care, which should include a holistic approach to care that is oriented toward the physical, psychological and social needs of older persons (48). Finally, public health initiatives to address modifiable risk factors, such as obesity, literacy, smoking and alcohol consumption, will help to prevent the occurrence of multimorbidity and thus reduce the health burden on older persons. Community-based healthy aging promotion programs, such as exercise classes, nutrition counseling, and smoking cessation programs, will play a vital role in promoting health and independence for older adults (49).

## Limitations

The limitations of this study are mainly reflected in the following four aspects: First, there was extremely high heterogeneity among the included studies, which represents the most prominent limitation of this study. Therefore, the reported overall pooled prevalence rate should be regarded as a rough average estimate, and its interpretation must be approached with extreme caution. Uncertain confounding factors-such as variations in survey methods, survey time points, and geographical diversity-may have contributed to these differences, and such uncertainties may introduce bias when results are combined. We attempted to explore potential sources of heterogeneity and concluded that differences in survey locations were the primary factor contributing to heterogeneity. Additionally, inconsistency in the definition of multimorbidity (e.g., most studies (*n* = 38) defined it as the presence of ≥2 chronic diseases, while the remaining 11 adopted a stricter threshold of ≥3 diseases) was another important reason, which directly led to incomparability among prevalence estimates. Second, the most significant limitation of this systematic review lies in the initial search strategy, which may have failed to comprehensively capture global evidence. Although we searched both Chinese and English databases, the relatively high proportion of studies from China among the finally included studies (26 from China vs. 23 from other countries worldwide) may not fully represent the global epidemiological status of multimorbidity. This imbalance may have been influenced by our original search strategy, which might have overrepresented terms commonly used in Chinese literature. Although we subsequently expanded the strategy to include more globally relevant terms, the existing evidence base in the literature could not be altered. This highlights a common challenge in global meta-analyses and suggests that the pooled prevalence should be interpreted primarily as a summary of existing identified literature rather than a definitive global average. The absence of certain key terms and the overrepresentation of Chinese studies indicate that our evidence base may be subject to geographical and linguistic biases. Thus, the results of this study should be considered an integration of available evidence from Chinese and English literature rather than a fully balanced global representation. Future research should intentionally ensure equitable geographical representation from the design stage onward. Third, in our main prevalence analysis, we included 49 studies, but only 17 of them were incorporated into the analysis of influencing factors. This limited sample size may reduce the statistical power to detect associations. Moreover, due to the inherent limitations of observational study designs, it is difficult to establish causal relationships between various risk factors and multimorbidity, which complicates the interpretation of the results. Fourth, many included studies did not report how missing data were handled, which may introduce selection bias and lead to underestimation of the true prevalence of multimorbidity. Additionally, publication bias may affect the pooled results, as studies with significant positive outcomes or larger sample sizes are more likely to be published, potentially skewing the overall findings. It is important to note that the lack of significant association between factors such as smoking, alcohol consumption, and physical inactivity with multimorbidity should be interpreted cautiously. This likely does not indicate a true absence of association but may rather reflect the limited number of original studies available for correlation analysis (*n* < 10), resulting in insufficient statistical power to detect a true effect. More specialized research is needed to deeply explore the complex relationship between these lifestyle factors and multimorbidity.

## Conclusion

In conclusion, this meta-analysis establishes that multimorbidity affects a substantial proportion of the world's older adult population, with a pooled prevalence of 46.0%. It is crucial to note that the evidence base of this study is more focused on reflecting the situation in China and regions where literature is widely published in Chinese and English. Consequently, while providing a global average, this figure conceals profound geographical and demographic disparities, underscoring that context-specific understanding, rather than a single estimate, is essential for effective policy. Beyond quantifying the burden, this study identifies a consistent set of associated factors, highlighting that advanced age and female sex are key non-modifiable risks, while obesity, lower education, economic disadvantage, and non-married status represent pivotal modifiable determinants rooted in social patterning. These findings compellingly argue for a dual approach: clinically, a shift toward integrated, patient-centered care models that address co-existing conditions; and at the policy level, multisectoral actions tackling social determinants of health to promote healthy aging and alleviate the burden of multimorbidity.

## Data Availability

The raw data supporting the conclusions of this article will be made available by the authors, without undue reservation.

## References

[1] Guo L. Effects of 24-form Tai Chi on cardio-pulmonary functions, exercise performances, and cognitive functions of the aged. Iran J Public Health. (2022) 51:2253–61. doi: 10.18502/ijph.v51i10.10983 36415801 PMC9647609

[2] Beard JR Officer A de Carvalho IA Sadana R Pot AM Michel JP . The world report on ageing and health: a policy framework for healthy ageing. Lancet. (2016) 387:2145–54. doi: 10.1016/S0140-6736(15)00516-4 26520231 PMC4848186

[3] Li J Han X Zhang X Wang S. Spatiotemporal evolution of global population ageing from 1960 to 2017. BMC Public Health. (2019) 19:127. doi: 10.1186/s12889-019-6465-2 30700286 PMC6354390

[4] Yang H Deng Q Geng Q Tang Y Ma J Ye W . Association of self-rated health with chronic disease, mental health symptom and social relationship in older people. Sci Rep. (2021) 11:14653. doi: 10.1038/s41598-021-94318-x 34282246 PMC8289838

[5] World Health Organization. The World Health Report 2008: Primary Health Care now More Than Ever (?2008). Geneva: World Health Organization Available online at: https://iris.who.int/handle/10665/43949

[6] Haque M Islam T Rahman NAA McKimm J Abdullah A Dhingra S. Strengthening primary health-care services to help prevent and control long-term (chronic) non-communicable diseases in low- and middle-income countries. Risk Manag Health Policy. (2020) 13:409–26. doi: 10.2147/RMHP.S239074 PMC7244358 32547272

[7] Blümel JE Carrillo-Larco RM Vallejo MS Chedraui P. Multimorbidity in a cohort of middle-aged women: risk factors and disease clustering. Maturitas. (2020) 137:45–9. doi: 10.1016/j.maturitas.2020.04.016 32498936 PMC7284304

[8] Zhou Z Shi M Liu M Gu J Silver Tarimo C Wu J . Multimorbidity in hospitalized patients admitted to general practice departments and its implications for the general practice healthcare system: a four-year longitudinal study in China. Front Public Health. (2021) 9:760792. doi: 10.3389/fpubh.2021.760792 34988048 PMC8720775

[9] Cong Z Huo M Jiang X Yu H. Factors associated with the level of self-management in elderly patients with chronic diseases: a pathway analysis. BMC Geriatr. (2024) 24:377. doi: 10.1186/s12877-024-04956-9 38671344 PMC11055297

[10] Ho IS Azcoaga-Lorenzo A Akbari A Davies J Hodgins P Khunti K . Variation in the estimated prevalence of multimorbidity: systematic review and meta-analysis of 193 international studies. BMJ Open. (2022) 12:e057017. doi: 10.1136/bmjopen-2021-057017 35487738 PMC9058768

[11] Nicholson K Liu W Fitzpatrick D Hardacre KA Roberts S Salerno J . Prevalence of multimorbidity and polypharmacy among adults and older adults: a systematic review. Lancet Healthy Longev. (2024) 5:e287–96. doi: 10.1016/S2666-7568(24)00007-2 38452787

[12] Roomaney RA van Wyk B Turawa EB. Pillay-van Wyk V. Multimorbidity in South Africa: a systematic review of prevalence studies. BMJ Open. (2021) 11:e048676. doi: 10.1136/bmjopen-2021-048676 PMC8496399 34615675

[13] Barnett K Mercer SW Norbury M Watt G Wyke S Guthrie B. Epidemiology of multimorbidity and implications for health care, research, and medical education: a cross-sectional study. Lancet. (2012) 380:37–43. doi: 10.1016/S0140-6736(12)60240-2 22579043

[14] Ding D Rogers K van der Ploeg H Stamatakis E Bauman AE. Traditional and emerging lifestyle risk behaviors and all-cause mortality in middle-aged and older adults: evidence from a large population-based Australian cohort. PLoS Med. (2015) 12:e1001917. doi: 10.1371/journal.pmed.1001917 26645683 PMC4672919

[15] Todd OM Burton JK Dodds RM Hollinghurst J Lyons RA Quinn TJ . New horizons in the use of routine data for ageing research. Age Ageing. (2020) 49:716–22. doi: 10.1093/ageing/afaa018 32043136 PMC7444666

[16] Chan SC Patrick Engksan J Jeevajothi Nathan J Sekhon JK Hussein N Suhaimi A . Developing a home-based pulmonary rehabilitation programme for patients with chronic respiratory diseases in Malaysia: a mixed-method feasibility study. J Glob Health. (2023) 13:04099. doi: 10.7189/jogh.13.04099 37883199 PMC10602205

[17] Zeng X Zhang Y Kwong JS Zhang C Li S Sun F . The methodological quality assessment tools for preclinical and clinical studies, systematic review and meta-analysis, and clinical practice guideline: a systematic review. J Evid Based Med. (2015) 8:2–10. doi: 10.1111/jebm.12141 25594108

[18] Li P Guan Y Zhou S Wang E Sun P Fei G . Mortality and risk factors for COVID-19 in hemodialysis patients: a systematic review and meta-analysis. Sci Prog. (2022) 105:368504221110858. doi: 10.1177/00368504221110858 35775141 PMC10358525

[19] Kim HR Choi CH Jo E A. Methodological quality assessment of meta-analysis studies in dance therapy using AMSTAR and AMSTAR 2. Healthcare. (2020) 8:446. doi: 10.3390/healthcare8040446 33139623 PMC7711445

[20] Savouré M Bousquet J Jaakkola JJK Jaakkola MS Jacquemin B Nadif R. Worldwide prevalence of rhinitis in adults: a review of definitions and temporal evolution. Clin Transl Allergy. (2022) 12:e12130. doi: 10.1002/clt2.12130 35344304 PMC8967272

[21] Ouyang W Guo G Xia J Zhao C Zhou X. Arthroscopic assisted versus open core decompression for osteonecrosis of the femoral head: a systematic review and meta-analysis. PLoS ONE. (2024) 19:e0313265. doi: 10.1371/journal.pone.0313265 39546449 PMC11567543

[22] Liu YT Chao JQ Wu XY Bao M Sheng MX Zhang BW. Prevalence and influencing factors of multiple chronic diseases among the elderly in Nanjing in 2019. Chin J Prev Med. (2022) 23:646–51. In Chinese. doi: 10.16506/j.1009-6639.2022.09.002

[23] Qi YT Liu Y Du J Liu YW Ma GF. Influencing factors of multimorbidity in the elderly in China based on health ecology model. Gen Pract Chin. (2023) 26:50–7. In Chinese. doi: 10.12114/j.issn.1007-9572.2022.0458

[24] Zhu PY Lei F Qiao B Ren XH. Prevalence and influencing factors of multiple chronic diseases among the elderly in Jinniu district of Chengdu in 2019. J Prev Med Inf. (2023) 39:84–92. In Chinese.

[25] Oliveira-Figueiredo DST Silva MPGPC Feitosa PYO Leite BC Rocha FL Andrade LDF. What is the burden of multimorbidity and the factors associated with its occurrence in elderly Brazilians? Rev Bras Enferm. (2024) 77:e20220809. doi: 10.1590/0034-7167-2022-0809 38716903 PMC11067935

[26] Reyes-Ortiz CA Lee T Campo-Arias A Ocampo-Chaparro JM Luque JS. Racial discrimination and multimorbidity among older adults in colombia: a national data analysis. Prev Chronic Dis. (2023) 20:E34. doi: 10.5888/pcd20.220360 37141184 PMC10159335

[27] Balakrishnan S Karmacharya I Ghimire S Mistry SK Singh DR Yadav OP . Prevalence of multimorbidity and its correlates among older adults in Eastern Nepal. BMC Geriatr. (2022) 22:425. doi: 10.1186/s12877-022-03115-2 35570271 PMC9109315

[28] Keomma K Bousquat A César CLG. Prevalence of multimorbidity in older adults in São Paulo, Brazil: a study with ISA-Capital. Rev Saude Publica. (2022) 56:69. doi: 10.11606/s1518-8787.2022056004252 35894406 PMC9337848

[29] Ha NT Le NH Khanal V Moorin R. Multimorbidity and its social determinants among older people in southern provinces, Vietnam. Int J Equity Health. (2015) 14:50. doi: 10.1186/s12939-015-0177-8 26024877 PMC4459670

[30] Bland JS. Age as a modifiable risk factor for chronic disease. Integr Med. (2018) 17:16–9. 31043904 PMC6469457

[31] Cribb L Hodge AM Yu C Li SX English DR Makalic E . Inflammation and epigenetic aging are largely independent markers of biological aging and mortality. J Gerontol A Biol Sci Med Sci. (2022) 77:2378–86. doi: 10.1093/gerona/glac147 35926479 PMC9799220

[32] Raghavachari N Wilmot B Dutta C. Optimizing translational research for exceptional health and life span: a systematic narrative of studies to identify translatable therapeutic target(s) for exceptional health span in humans. J Gerontol A Biol Sci Med Sci. (2022) 77:2272–80. doi: 10.1093/gerona/glac065 35279027 PMC9678194

[33] Pastorino S Richards M Pierce M Ambrosini GL. A high-fat, high-glycaemic index, low-fibre dietary pattern is prospectively associated with type 2 diabetes in a British birth cohort. Br J Nutr. (2016) 115:1632–42. doi: 10.1017/S0007114516000672 27245103 PMC4907349

[34] Rossaneis MA Haddad Mdo C Mathias TA Marcon SS. Differences in foot self-care and lifestyle between men and women with diabetes mellitus. Rev Lat Am Enfermagem. (2016) 24:e2761. doi: 10.1590/1518-8345.1203.2761 27533270 PMC4996089

[35] Assari S Bazargan M. Race/ethnicity, socioeconomic status, and polypharmacy among older Americans. Pharmacy. (2019) 7:41. doi: 10.3390/pharmacy7020041 31027176 PMC6631748

[36] Chen YT Ruan WQ ZHANG LL Huang JH Liu FY Liu XJ. Prevalence of chronic diseases in Hakka elderly in Fujian province: differences in main demographic characteristics. J Naval Med Univ. (2023) 44:583–88. In Chinese. doi: 10.16781/j.CN31-2187/R.20220466

[37] Guo D Jin CG Xu YB Liang XY. Association between body mass index and comorbidity in the elderly aged 65 years and over. Chronic Dis Prev Control Chin. (2022) 30:129–33. In Chinese. doi: 10.16386/j.cjpccd.issn.1004-6194.2022.02.011

[38] Medina C Janssen I Campos I Barquera S. Physical inactivity prevalence and trends among Mexican adults: results from the National Health and Nutrition survey (ENSANUT) 2006 and 2012. BMC Public Health. (2013) 13:1063. doi: 10.1186/1471-2458-13-1063 24215173 PMC3883516

[39] Ghanem AS Nguyen CM Mansour Y Fábián G Rusinné Fedor A Nagy A . Investigating the association between sociodemographic factors and chronic disease risk in adults aged 50 and above in the Hungarian population. Healthcare. (2023) 11:1940. doi: 10.3390/healthcare11131940 37444774 PMC10340376

[40] Tao S Sun S Wu S Peng T Cao L Yan M . Current status and influencing factors of health literacy among older adults in combined medical and nursing care institutions: a cross-sectional study. Front Public Health. (2024) 11:1323335. doi: 10.3389/fpubh.2023.1323335 38292383 PMC10825950

[41] Tian Y Zhan Y Wu M. Gender differences in migrant workers health in China. Int J Public Health. (2023) 68:1605018. doi: 10.3389/ijph.2023.1605018 37655264 PMC10467421

[42] Su W Lin Y Yang L Zhang W Dong Z Zhang J. Prevalence and influencing factors of chronic diseases among the elderly in Southwest China: a cross-sectional study based on community in urban and rural areas. Prev Med Rep. (2024) 44:102799. doi: 10.1016/j.pmedr.2024.102799 39045092 PMC11263618

[43] Wang J Zhou Y Zhang Q Li J Zhai D Li J. et al. Loneliness among older Chinese individuals: the status quo and relationships with activity-related factors [published correction appears in *BMC Geriatr*]. (2024) 24:270. doi: 10.1186/s12877-024-04821-9 PMC10953144 38504165

[44] Chia F Huang WY Huang H Wu CE. Promoting healthy behaviors in older adults to optimize health-promoting lifestyle: an intervention study. Int J Environ Res Public Health. (2023) 20:1628. doi: 10.3390/ijerph20021628 36674395 PMC9866478

[45] Schaffler J Leung K Tremblay S Merdsoy L Belzile E Lambrou A . The effectiveness of self-management interventions for individuals with low health literacy and/or low income: a descriptive systematic review. J Gen Intern Med. (2018) 33:510–23. doi: 10.1007/s11606-017-4265-x 29427178 PMC5880764

[46] Wang Y Wu XY Wang HHX Li YT Fu Y Wang JJ . Body constitution and unhealthy lifestyles in a primary care population at high cardiovascular risk: new insights for health management. Int J Gen Med. (2021) 14:6991–7001. doi: 10.2147/IJGM.S329321 34707390 PMC8544129

[47] Nekouei Marvi Langari M Lindström J Heponiemi T Kaihlanen AM Hietapakka L Heidarian Miri H . Integrated care competencies and their association with cross-cultural competence among registered nurses: a cross-sectional questionnaire survey. Nurs Open. (2024) 11:e2062. doi: 10.1002/nop2.2062 38268264 PMC10840592

[48] Dostálová V Bártová A Bláhová H Holmerová I. The experiences and needs of frail older people receiving home health care: A qualitative study. Int J Older People Nurs. (2022) 17:e12418. doi: 10.1111/opn.12418 34418315 PMC9285561

[49] Kim C Sung J Han JY Jee S Lee JW Lee JH . Evaluation of current resources available for community-based cardiac rehabilitation in korea: a nationwide survey study. J Korean Med Sci. (2022) 37:e109. doi: 10.3346/jkms.2022.37.e109 35411729 PMC9001186

[50] Kong Y Wang L Yang GH Chen M Wang SR. Current status and multimorbidity patterns of chronic diseases in the elderly with registered medical records in Guizhou province. Mod Prev Med. (2021) 48:3216–9. In Chinese. doi: 10.20043/j.cnki.mpm.2021.17.033

[51] Li X Cai L Wang XM Cui WL Lv SQ He JH. Prevalence of five common chronic diseases and multimorbidity in rural elderly in Yunnan province and its relationship with socioeconomic status. Chin J Dis Control. (2019) 23:630–4. In Chinese. doi: 10.16462/j.cnki.zhjbkz.2019.06.003

[52] Li GX Liu AQ Zhou JG Jia HB Yuan TT Zhang H . Influencing factors of common chronic diseases in the elderly with home medical and nursing care in community. J Zhengzhou Univ (Medical Edition). (2024) 59:325–30. In Chinese. doi: 10.13705/j.issn.1671-6825.2023.06.045

[53] Liu XX Guo HJ Yu YK Chen ZX Wang Q Liu JL . Current status and association rules of multiple chronic diseases in the elderly in Mianyang city. J Prev Med Inf . (2023) 39:52–9. In Chinese.

[54] Tian L Wang L Wu DK Bo F Wang XN An N . Analysis of risk factors for 20 diseases in the elderly aged 65 years and over in Heilongjiang province. Chin Public Health Adm. (2023) 39:897–901. In Chinese. doi: 10.19568/j.cnki.23-1318.2023.06.0037

[55] Zhang XQ Liu YY. Study on the relationship between multimorbidity and self-rated health among the elderly in a rural area of northwest China. Chin Med J. (2024) 59:538–44. In Chinese. doi: 10.3969/j.issn.1008-1070.2024.05.018

[56] Zhang H Qi SG Li ZX Dong Z Wang ZH. Prevalence of common chronic diseases among the elderly in communities of six provinces and cities in 2015. Public Health. (2019) 13:122–25. In Chinese. doi: 10.16760/j.cnki.sdggws.2019.03.002

[57] Cao M Li Y Huang JY Gu Q Zhang TT Tian QF. Investigation on the prevalence of chronic diseases and multimorbidity among the elderly in medical and nursing institutions in Henan Province. J Zhengzhou Univ (Medical Edition). (2021) 56:800–4. In Chinese. doi: 10.13705/j.issn.1671-6825.2020.11.110

[58] Liu DN Zheng Y Liu XX Yang QD Su QY Wu H . Prevalence of multiple chronic diseases among elderly residents in Shanghai communities. Jiangsu Prev Med. (2023) 34:24–7. In Chinese. doi: 10.13668/j.issn.1006-9070.2023.01.006

[59] Yu ZJ Hu J Dai T. Analysis of the Current Situation and Population Distribution Characteristics of Chronic Comorbidities Among Elderly Residents in Shangang Village Zhangjiagang City. Jiangsu Health Serv Adm. (2023) 34:839–44. In Chinese.

[60] Mu YJ Xu S Chen HE Dai SH Zhang J Liu YX . Prevalence of multiple chronic diseases and its impact on physical function among the elderly in Nanshan District of Shenzhen City. Public health China. (2023) 39:982–5. In Chinese. doi: 10.11847/zgggws1140719

[61] Wang WH Ji DK Xue CH Guo HJ Xu JS Wu YX . Prevalence of multiple chronic diseases in the elderly in Jiangsu province in 2021. Public Health China. (2024) 40:450–5. In Chinese. doi: 10.11847/zgggws1143201

[62] Yao YL Hu QQ Shi WL Feng X Huang WB Duan GY . Analysis of the status of common chronic diseases in the elderly in Guancheng Hui community of Zhengzhou. Henan Med Res. (2022) 31:1930–3. In Chinese. doi: 10.3969/j.issn.1004-437X.2022.11.003

[63] Zhou FK Tan W Liu D Cheng GR Xu L Lian PF . Prevalence of dementia and its association with multimorbidity in the elderly aged 65 years and over in communities of Hubei Province. Chin J Dis Control. (2023) 27:627–32. In Chinese. doi: 10.16462/j.cnki.zhjbkz.2023.06.002

[64] Ko S Park S Kim J Subramanian SV Kim R. Spousal multimorbidity and depressive symptoms among older Indian couples: do one's own health status and sex matter? Geroscience. (2024) 46:885–96. doi: 10.1007/s11357-023-00822-5 37233884 PMC10828161

[65] Kohler S Bärnighausen T Kazonda P Leyna GH Lohmann J Killewo J . Chronic conditions and multimorbidity among middle-aged and elderly peri-urban dwellers in dar es Salaam, Tanzania. Int J Public Health. (2024) 69:1606387. doi: 10.3389/ijph.2024.1606387 38988502 PMC11233465

[66] Lee C Park YH Cho B Lee HA. A network-based approach to explore comorbidity patterns among community-dwelling older adults living alone. Geroscience. (2024) 46:2253–64. doi: 10.1007/s11357-023-00987-z 37924440 PMC10828172

[67] Maimaitiwusiman Z Wumaier A Xiao W Xuekelati S Halan B Xiang H . Ethnic and geographic variations in multiple chronic conditions among community-dwelling older people in Xinjiang: a cross-sectional study. BMC Geriatr. (2023) 23:455. doi: 10.1186/s12877-023-04159-8 37488530 PMC10367248

[68] You L Guo L Li N Zhong J Er Y Zhao M. Association between multimorbidity and falls and fear of falling among older adults in eastern China: a cross-sectional study. Front Public Health. (2023) 11:1146899. doi: 10.3389/fpubh.2023.1146899 37275486 PMC10234124

[69] Yang K Yang S Chen Y Cao G Xu R Jia X . Multimorbidity patterns and associations with gait, balance and lower extremity muscle function in the elderly: a cross-sectional study in northwest China. Int J Gen Med. (2023) 16:3179–92. doi: 10.2147/IJGM.S418015 37533839 PMC10392815

[70] Honda Y Nakamura M Aoki T Ojima T. Multimorbidity patterns and the relation to self-rated health among older Japanese people: a nationwide cross-sectional study. BMJ Open. (2022) 12:e063729. doi: 10.1136/bmjopen-2022-063729 36538382 PMC9438194

[71] Lynch DH Petersen CL Fanous MM Spangler HB Kahkoska AR Jimenez D . The relationship between multimorbidity, obesity and functional impairment in older adults. J Am Geriatr Soc. (2022) 70:1442–9. doi: 10.1111/jgs.17683 35113453 PMC9106850

[72] Shariff Ghazali S Seman Z Zainuddin NH Omar MA Sooryanarayana R Ariaratnam S . Prevalence and factors associated with multimorbidity among older adults in Malaysia: a population-based cross-sectional study. BMJ Open. (2021) 11:e052126. doi: 10.1136/bmjopen-2021-052126 34670764 PMC8529977

[73] Lin WQ Yuan LX Sun MY Wang C Liang EM Li YH . Prevalence and patterns of multimorbidity in chronic diseases in Guangzhou, China: a data mining study in the residents' health records system among 31 708 community-dwelling elderly people. BMJ Open. (2022) 12:e056135. doi: 10.1136/bmjopen-2021-056135 35613781 PMC9134174

[74] Sara HH Chowdhury MAB Haque MA. Multimorbidity among elderly in Bangladesh. Aging Med. (2018) 1:267–75. doi: 10.1002/agm2.12047 31942503 PMC6880734

[75] Smith L Shin JI Haro JM Jacob L López Sánchez GF Tully MA . Physical multimorbidity and wish to die among adults aged ≥65 years: a cross-sectional analysis of the Irish Longitudinal Study on ageing. J Affect Disord. (2022) 313:263–9. doi: 10.1016/j.jad.2022.06.063 35764230

[76] Jovic D Vukovic D Marinkovic J. Prevalence and patterns of multi-morbidity in serbian adults: a cross-sectional study. PLoS ONE. (2016) 11:e0148646. doi: 10.1371/journal.pone.0148646 26871936 PMC4752477

[77] Puth MT Weckbecker K Schmid M Münster E. Prevalence of multimorbidity in Germany: impact of age and educational level in a cross-sectional study on 19,294 adults. BMC Public Health. (2017) 17:826. doi: 10.1186/s12889-017-4833-3 29047341 PMC5648462

[78] Bähler C Huber CA Brüngger B Reich O. Multimorbidity, health care utilization and costs in an elderly community-dwelling population: a claims data based observational study. BMC Health Serv Res. (2015) 15:23. doi: 10.1186/s12913-015-0698-2 25609174 PMC4307623

[79] Yadav UN Ghimire S Mistry SK Shanmuganathan S Rawal LB Harris M. Prevalence of non-communicable chronic conditions, multimorbidity and its correlates among older adults in rural Nepal: a cross-sectional study. BMJ Open. (2021) 11:e041728. doi: 10.1136/bmjopen-2020-041728 33632751 PMC7908905

[80] Hien H Berthé A Drabo MK Meda N Konaté B Tou F . Prevalence and patterns of multimorbidity among the elderly in Burkina Faso: cross-sectional study. Trop Med Int Health. (2014) 19:1328–33. doi: 10.1111/tmi.12377 25164626

[81] Asante D Rio J Stanaway F Worley P Isaac V. Psychological distress, multimorbidity and health services among older adults in rural South Australia. J Affect Disord. (2022) 309:453–60. doi: 10.1016/j.jad.2022.04.140 35490879

[82] Abdulazeez ZU Grema BA Michael GC Abdulkadir Z. Multimorbidity and functional status of the elderly in a primary care setting of northern Nigeria: a cross-sectional study. West Afr J Med. (2021) 38:620–8. 34330282

[83] Nugraha S Hapsari I Sabarinah Pengpid S Peltzer K. Multimorbidity Increases the risk of falling among indonesian elderly living in community dwelling and elderly home: a cross sectional study. Indian J Public Health Res Dev. (2019) 10:2263–7. doi: 10.5958/0976-5506.2019.03898.1

[84] He YZ Yu JQ Zheng JZ Tong Y. Association of health promotion behaviors and multimorbidity in the elderly in Ningxia. Chin Gen Pract. (2023) 28:3526–32. doi: 10.12114/j.issn.1007-9572.2023.0026

[85] Hou YT Jiang DD Liu XJ He MK Mao ZF. Prevalence and related factors of comorbidity of chronic diseases among community elderly in Wuhan city. Chin J Public Health. (2020) 11:1604–7. doi: 10.11847/zgggws1122572

[86] Marzban M Jamshidi A Khorrami Z Hall M Batty JA Farhadi A . Determinants of multimorbidity in older adults in Iran: a cross-sectional study using latent class analysis on the Bushehr Elderly Health (BEH) program. BMC Geriatr. (2024) 24:247. doi: 10.1186/s12877-024-04848-y 38468227 PMC10929162

[87] Aye SKK Hlaing HH Htay SS Cumming R. Multimorbidity and health seeking behaviours among older people in Myanmar: A community survey. PLoS One. (2019) 14:e0219543. doi: 10.1371/journal.pone.0219543 31295287 PMC6622547

