# Supplementary material for: About half of older adults have two or more chronic conditions at the same time: a systematic review and meta-analysis
Source: Front Public Health. 2025 Dec 11;13:1680745. doi: 10.3389/fpubh.2025.1680745 (PMC12738304; doi:10.3389/fpubh.2025.1680745)
Supplement: Supplementary file 1 [file Table_1.doc]

| Supplementary Table 1. PRISMA 2020 Checklist | | | |
| --- | --- | --- | --- |
| **Section and Topic** | **Item #** | **Checklist item** | **Location where item is reported** |
| **TITLE** | | |  |
| Title | 1 | Identify the report as a systematic review. | Page 1 |
| **ABSTRACT** | | |  |
| Abstract | 2 | See the PRISMA 2020 for Abstracts checklist. | Page 1-2 |
| **INTRODUCTION** | | |  |
| Rationale | 3 | Describe the rationale for the review in the context of existing knowledge. | Page 2 |
| Objectives | 4 | Provide an explicit statement of the objective(s) or question(s) the review addresses. | Page 2 |
| **METHODS** | | |  |
| Eligibility criteria | 5 | Specify the inclusion and exclusion criteria for the review and how studies were grouped for the syntheses. | Page 3 |
| Information sources | 6 | Specify all databases, registers, websites, organisations, reference lists and other sources searched or consulted to identify studies. Specify the date when each source was last searched or consulted. | Page 2-3 |
| Search strategy | 7 | Present the full search strategies for all databases, registers and websites, including any filters and limits used. | Page 2-3, Supplementary Table 2 |
| Selection process | 8 | Specify the methods used to decide whether a study met the inclusion criteria of the review, including how many reviewers screened each record and each report retrieved, whether they worked independently, and if applicable, details of automation tools used in the process. | Page 3 |
| Data collection process | 9 | Specify the methods used to collect data from reports, including how many reviewers collected data from each report, whether they worked independently, any processes for obtaining or confirming data from study investigators, and if applicable, details of automation tools used in the process. | Page 3-4 |
| Data items | 10a | List and define all outcomes for which data were sought. Specify whether all results that were compatible with each outcome domain in each study were sought (e.g. for all measures, time points, analyses), and if not, the methods used to decide which results to collect. | Page 3 |
|  | 10b | List and define all other variables for which data were sought (e.g. participant and intervention characteristics, funding sources). Describe any assumptions made about any missing or unclear information. | Page 3 |
| Study risk of bias assessment | 11 | Specify the methods used to assess risk of bias in the included studies, including details of the tool(s) used, how many reviewers assessed each study and whether they worked independently, and if applicable, details of automation tools used in the process. | Page 3 |
| Effect measures | 12 | Specify for each outcome the effect measure(s) (e.g. risk ratio, mean difference) used in the synthesis or presentation of results. | Page 4 |
| Synthesis methods | 13a | Describe the processes used to decide which studies were eligible for each synthesis (e.g. tabulating the study intervention characteristics and comparing against the planned groups for each synthesis (item #5)). | Page 3-4 |
|  | 13b | Describe any methods required to prepare the data for presentation or synthesis, such as handling of missing summary statistics, or data conversions. | Page 3-4 |
|  | 13c | Describe any methods used to tabulate or visually display results of individual studies and syntheses. | Page 4 |
|  | 13d | Describe any methods used to synthesize results and provide a rationale for the choice(s). If meta-analysis was performed, describe the model(s), method(s) to identify the presence and extent of statistical heterogeneity, and software package(s) used. | Page 4 |
|  | 13e | Describe any methods used to explore possible causes of heterogeneity among study results (e.g. subgroup analysis, meta-regression). | Page 4 |
|  | 13f | Describe any sensitivity analyses conducted to assess robustness of the synthesized results. | Page 4 |
| Reporting bias assessment | 14 | Describe any methods used to assess risk of bias due to missing results in a synthesis (arising from reporting biases). | Page 4 |
| Certainty assessment | 15 | Describe any methods used to assess certainty (or confidence) in the body of evidence for an outcome. | Page 3-4 |
| **RESULTS** | | |  |
| Study selection | 16a | Describe the results of the search and selection process, from the number of records identified in the search to the number of studies included in the review, ideally using a flow diagram. | Page 4, Fig.1 |
|  | 16b | Cite studies that might appear to meet the inclusion criteria, but which were excluded, and explain why they were excluded. | Page 4 |
| Study characteristics | 17 | Cite each included study and present its characteristics. | Page 4, Table 1 |
| Risk of bias in studies | 18 | Present assessments of risk of bias for each included study. | Page 4, Supplementary Table 3 |
| Results of individual studies | 19 | For all outcomes, present, for each study: (a) summary statistics for each group (where appropriate) and (b) an effect estimate and its precision (e.g. confidence/credible interval), ideally using structured tables or plots. | Page 4-5, Fig.2 |
| Results of syntheses | 20a | For each synthesis, briefly summarise the characteristics and risk of bias among contributing studies. | Page 4-5 |
|  | 20b | Present results of all statistical syntheses conducted. If meta-analysis was done, present for each the summary estimate and its precision (e.g. confidence/credible interval) and measures of statistical heterogeneity. If comparing groups, describe the direction of the effect. | Page 5,10, Table 2, Table 3 |
|  | 20c | Present results of all investigations of possible causes of heterogeneity among study results. | Page 5,9-10 |
|  | 20d | Present results of all sensitivity analyses conducted to assess the robustness of the synthesized results. | Page 5,10 |
| Reporting biases | 21 | Present assessments of risk of bias due to missing results (arising from reporting biases) for each synthesis assessed. | Page 5,10-11 |
| Certainty of evidence | 22 | Present assessments of certainty (or confidence) in the body of evidence for each outcome assessed. | Page 5,10-11, Fig.4 |
| **DISCUSSION** | | |  |
| Discussion | 23a | Provide a general interpretation of the results in the context of other evidence. | Page 11-13 |
|  | 23b | Discuss any limitations of the evidence included in the review. | Page 13-14 |
|  | 23c | Discuss any limitations of the review processes used. | Page 13-14 |
|  | 23d | Discuss implications of the results for practice, policy, and future research. | Page 12-13 |
| **OTHER INFORMATION** | | |  |
| Registration and protocol | 24a | Provide registration information for the review, including register name and registration number, or state that the review was not registered. | Page 2 |
|  | 24b | Indicate where the review protocol can be accessed, or state that a protocol was not prepared. | Page 2 |
|  | 24c | Describe and explain any amendments to information provided at registration or in the protocol. | / |
| Support | 25 | Describe sources of financial or non-financial support for the review, and the role of the funders or sponsors in the review. | Page 14 |
| Competing interests | 26 | Declare any competing interests of review authors. | Page 14 |
| Availability of data, code and other materials | 27 | Report which of the following are publicly available and where they can be found: template data collection forms; data extracted from included studies; data used for all analyses; analytic code; any other materials used in the review. | Page 14 |

*From:*  Page MJ, McKenzie JE, Bossuyt PM, Boutron I, Hoffmann TC, Mulrow CD, et al. The PRISMA 2020 statement: an updated guideline for reporting systematic reviews. BMJ 2021;372:n71. doi: 10.1136/bmj.n71. This work is licensed under CC BY 4.0. To view a copy of this license, visit <https://creativecommons.org/licenses/by/4.0/>

Supplementary Table 2. Search Strategy

| **Databases [Platform]** | **Results** |
| --- | --- |
| PubMed (*July 30, 2025*) | 13083 |
| Web of Science (*July 30, 2025*) | 2909 |
| Cochrane Library (*July 30, 2025*) | 1146 |
| Embase (*July 30, 2025*) | 11041 |
| CNKI (*July 30, 2025*) | 548 |
| WANFANG (*July 30, 2025*) | 111 |
| CBM (*July 30, 2025*) | 106 |
| VIP (*July 30, 2025*) | 198 |
| TOTAL | 29142 |
| After Removing Duplications | **22729** |

| **Databases** | **Set** | **Search terms** | **Items found** |
| --- | --- | --- | --- |
| PubMed | 1 | (aged[MeSH Terms] OR frail elderly[MeSH Terms] OR older adults[MeSH Terms]) | 3727549 |
|  | 2 | ("elder*"[Title/Abstract] OR "adult*"[Title/Abstract] OR "senior citizen"[Title/Abstract] OR "older"[Title/Abstract]) | 2444721 |
|  | 3 | 1 OR 2 | 5456204 |
|  | 4 | (comorbidity[MeSH Terms] OR multimorbidity[MeSH Terms] OR multiple chronic conditions[MeSH Terms]) | 135735 |
|  | 5 | ("multiple chronic condition*"[Title/Abstract] OR "multiple chronic illnesses"[Title/Abstract] OR "multiple chronic health conditions"[Title/Abstract] OR "multiple chronic medical conditions"[Title/Abstract] OR "concurrent chronic illnesses"[Title/Abstract] OR "multiple chronic diseases"[Title/Abstract] OR "multiple chronic disorders"[Title/Abstract] OR "concurrent chronic conditions"[Title/Abstract] OR "concurrent chronic diseases"[Title/Abstract] OR "concurrent chronic medical conditions"[Title/Abstract] OR "concurrent chronic health conditions"[Title/Abstract]) | 3310 |
|  | 6 | 4 OR 5 | 137975 |
|  | 7 | (prevalence[MeSH Terms] OR morbidity[MeSH Terms] OR incidence[MeSH Terms] OR epidemiology[MeSH Terms]) | 728379 |
|  | 8 | ("Prevalence*"[Title/Abstract] OR "Point Prevalence*"[Title/Abstract] OR "Social Epidemiolog*"[Title/Abstract] OR "Period Prevalence*"[Title/Abstract] OR "Attack Rate*"[Title/Abstract] OR "epidemiologic factors"[Title/Abstract] OR "Incidence*"[Title/Abstract]) | 1892996 |
|  | 9 | 7 OR 8 | 1485912 |
|  | 10 | ("Prevalence*"[Title/Abstract] OR "Point Prevalence*"[Title/Abstract] OR "Social Epidemiolog*"[Title/Abstract] OR "Period Prevalence*"[Title/Abstract] OR "Attack Rate*"[Title/Abstract] OR "epidemiologic factors"[Title/Abstract] OR "Incidence*"[Title/Abstract]) | 3917416 |
|  | 11 | ("factor*"[Title/Abstract] OR "risk factor score*"[Title/Abstract] OR "social risk factor*"[Title/Abstract] OR "relative risk*"[Title/Abstract] OR "risk*"[Title/Abstract] OR "protective factor*"[Title/Abstract] OR "associated factors"[Title/Abstract] OR "population* at risk"[Title/Abstract]) | 6862824 |
|  | 12 | 10 OR 11 | **7275105** |
|  | 13 | (epidemiologic studies[MeSH Terms] OR cross-sectional studies[MeSH Terms]) | 3504411 |
|  | 14 | ("cross-sectional design"[Title/Abstract] OR "cross-sectional research"[Title/Abstract] OR "cross sectional analys*"[Title/Abstract] OR "cross-sectional stud*"[Title/Abstract] OR "clinical epidemiology"[Title/Abstract] OR "disease frequency survey*"[Title/Abstract] OR "epidemiologic study"[Title/Abstract] OR "prevalence stud*"[Title/Abstract] OR "cross sectional survey*"[Title/Abstract] OR "epidemiological stud*"[Title/Abstract]) | 526622 |
|  | 15 | 13 OR 14 | 3696221 |
|  | 16 | 3AND6 AND9 AND 12AND 15 | **13083** |
| Web of Science | 1 | TS=("aged" OR "frail elderly" OR "older adults" OR "elder*" OR "adult*" OR "senior citizen" OR "older") | 3601335 |
|  | 2 | TS=("comorbidity" OR "multimorbidity" OR "multiple chronic conditions" OR "multiple chronic condition*" OR "multiple chronic illnesses" OR "multiple chronic health conditions" OR "multiple chronic medical conditions" OR "concurrent chronic illnesses" OR "multiple chronic diseases" OR "multiple chronic disorders" OR "concurrent chronic conditions" OR "concurrent chronic diseases" OR "concurrent chronic medical conditions" OR "concurrent chronic health conditions") | 148164 |
|  | 3 | TS=("morbidity" OR "incidence" OR "epidemiology" OR "Prevalence*" OR "Point Prevalence*" OR "Social Epidemiolog*" OR "Period Prevalence*" OR "Attack Rate*" OR "epidemiologic factors" OR "Incidence*") | 3016366 |
|  | 4 | TS=("risk factors" OR "risk" OR" influencing factor" OR "protection" OR "factor*" OR "risk factor score*" OR "social risk factor*" OR "relative risk*" OR "risk*" OR "protective factor*" OR "associated factors" OR "population* at risk") | 11741623 |
|  | 5 | TS=("epidemiologic studies" OR "cross-sectional studies" OR "cross-sectional design" OR "cross-sectional research" OR "cross sectional analys*" OR "cross-sectional stud*" OR "clinical epidemiology" OR "disease frequency survey*" OR "epidemiologic study" OR "prevalence stud*" OR "cross sectional survey*" OR "epidemiological stud*") | 567864 |
|  | 6 | 1 AND 2 AND 3 AND 4 AND 5 | **2909** |
| Cochrane Library | 1 | MeSH descriptor: [Aged] explode all trees | 281610 |
|  | 2 | MeSH descriptor: [Frail Elderly] explode all trees | 1201 |
|  | 3 | (older adults):ti,ab,kw OR (elder*):ti,ab,kw OR (adult*):ti,ab,kw OR (senior citizen):ti,ab,kw OR (older):ti,ab,kw | 976972 |
|  | 4 | 1 OR 2 OR 3 | 1046514 |
|  | 5 | MeSH descriptor: [Comorbidity] explode all trees | 5238 |
|  | 6 | MeSH descriptor: [Multimorbidity] explode all trees | 180 |
|  | 7 | MeSH descriptor: [Multiple Chronic Conditions] explode all trees | 97 |
|  | 8 | (multiple chronic condition*):ti,ab,kw OR (multiple chronic illnesses):ti,ab,kw OR (multiple chronic health conditions):ti,ab,kw OR (multiple chronic medical conditions):ti,ab,kw OR (concurrent chronic illnesses):ti,ab,kw OR (multiple chronic diseases):ti,ab,kw OR (multiple chronic disorders):ti,ab,kw OR (concurrent chronic conditions):ti,ab,kw OR (concurrent chronic diseases):ti,ab,kw OR (concurrent chronic medical conditions):ti,ab,kw OR (concurrent chronic health conditions):ti,ab,kw | 6103 |
|  | 9 | 5 OR 6 OR 7 OR 8 | 11147 |
|  | 10 | MeSH descriptor: [Risk Factors] explode all trees | 38308 |
|  | 11 | MeSH descriptor: [Risk] explode all trees | 56620 |
|  | 12 | MeSH descriptor: [Protective Factors] explode all trees | 286 |
|  | 13 | (Prevalences):ti,ab,kw OR (Incidences):ti,ab,kw OR (epidemics):ti,ab,kw OR (epidemics):ti,ab,kw OR (Frequency):ti,ab,kw OR (Associated factors):ti,ab,kw OR (Risk Factor):ti,ab,kw OR (Relative Risk):ti,ab,kw OR (Protective Factor):ti,ab,kw | 242678 |
|  | 14 | 6 OR 7 OR 8 OR 9 OR 10 OR 11 OR 12 OR 13 | 281653 |
|  | 15 | MeSH descriptor: [Prevalence] explode all trees | 7324 |
|  | 16 | MeSH descriptor: [Morbidity] explode all trees | 21791 |
|  | 17 | MeSH descriptor: [Incidence] explode all trees | 14178 |
|  | 18 | MeSH descriptor: [Epidemiology] explode all trees | 103 |
|  | 19 | (Prevalence*):ti,ab,kw OR (Point Prevalence*):ti,ab,kw OR (Social Epidemiolog*):ti,ab,kw OR (Period Prevalence*):ti,ab,kw OR (Attack Rate*):ti,ab,kw OR (epidemiologic factors):ti,ab,kw OR (Incidence*):ti,ab,kw | 218949 |
|  | 20 | 15 OR 16 OR 17 OR 18 OR 19 | 29822 |
|  | 21 | MeSH descriptor: [Risk Factors] explode all trees | 37977 |
|  | 22 | MeSH descriptor: [Risk] explode all trees | 55816 |
|  | 23 | MeSH descriptor: [Protective Factors] explode all trees | 276 |
|  | 24 | (factor*):ti,ab,kw OR (risk factor score*):ti,ab,kw OR (social risk factor*):ti,ab,kw OR (relative risk*):ti,ab,kw OR (risk*):ti,ab,kw OR (protective factor*):ti,ab,kw OR (associated factors):ti,ab,kw OR (population* at risk):ti,ab,kw | 543817 |
|  | 25 | 21 OR 22 OR 23 OR 24 | 546250 |
|  | 26 | MeSH descriptor: [Epidemiologic Studies] explode all trees | 223094 |
|  | 27 | MeSH descriptor: [Cross-Sectional Studies] explode all trees | 10044 |
|  | 28 | (cross-sectional design):ti,ab,kw OR (cross-sectional research):ti,ab,kw OR (cross sectional analys*):ti,ab,kw OR (cross-sectional stud*):ti,ab,kw OR (clinical epidemiology):ti,ab,kw OR (disease frequency survey*):ti,ab,kw OR (epidemiologic study):ti,ab,kw OR (prevalence stud*):ti,ab,kw OR (cross sectional survey*):ti,ab,kw OR (epidemiological stud*):ti,ab,kw | 111320 |
|  | 29 | 26 OR 27 OR 28 | 301829 |
|  | 30 | 4 AND 9 AND 14 AND 20 AND 25 | **1146** |
| Embase | 1 | ('aged'/exp OR 'elderly'/exp OR 'frail elderly'/exp OR 'elder*':ab,ti OR 'senior citizen*':ab,ti OR 'older adult*':ab,ti OR 'older':ab,ti) | 5003986 |
|  | 2 | ('comorbidity'/exp OR 'multimorbidity'/exp OR 'multiple chronic condition*':ab,ti OR 'multiple chronic illness*':ab,ti OR 'multiple chronic health condition*':ab,ti OR 'multiple chronic medical condition*':ab,ti OR 'concurrent chronic illness*':ab,ti OR 'multiple chronic disease*':ab,ti OR 'multiple chronic disorder*':ab,ti OR 'concurrent chronic condition*':ab,ti OR 'concurrent chronic disease*':ab,ti OR 'concurrent chronic medical condition*':ab,ti OR 'concurrent chronic health condition*':ab,ti) | 492511 |
|  | 3 | ('prevalence'/exp OR 'incidence'/exp OR 'morbidity'/exp OR 'epidemiology'/exp OR 'prevalence*':ab,ti OR 'point prevalence*':ab,ti OR 'period prevalence*':ab,ti OR 'attack rate*':ab,ti OR 'epidemiologic factor*':ab,ti OR 'incidence*':ab,ti OR 'social epidemiolog*':ab,ti) | 6365559 |
|  | 4 | ('risk factor'/exp OR 'risk'/exp OR 'factor*':ab,ti OR 'risk factor score*':ab,ti OR 'social risk factor*':ab,ti OR 'relative risk*':ab,ti OR 'risk*':ab,ti OR 'protective factor*':ab,ti OR 'associated factor*':ab,ti OR 'population* at risk':ab,ti) | 10031938 |
|  | 5 | ('cross-sectional study'/exp OR 'epidemiologic study'/exp OR 'cross-sectional design':ab,ti OR 'cross-sectional research':ab,ti OR 'cross sectional analys*':ab,ti OR 'cross-sectional stud*':ab,ti OR 'clinical epidemiology':ab,ti OR 'disease frequency survey*':ab,ti OR 'epidemiologic stud*':ab,ti OR 'prevalence stud*':ab,ti OR 'cross sectional survey*':ab,ti OR 'epidemiological stud*':ab,ti) | 980575 |
|  | 6 | 1 AND 2 AND 3 AND 4 AND 5 | **11041** |
| CNKI |  | 主题 = (老年人 OR 老年 OR 老人) AND主题 = (共病 OR 多病共存 OR 多重慢性病 OR 慢性病共病 OR 多种慢性疾病 OR 共存疾病 OR 慢性病多病共存 OR 慢病共病) AND主题 = (患病率 OR 发病率 OR 患病情况) AND主题 = (影响因素 OR 相关因素 OR 保护因素 OR 风险因素 OR 危险因素) AND 主题 = (横断面研究 OR 横断面调查 OR 现况调查 OR 流行病学研究 OR 流行病学调查) | **548** |
| WANFANG |  | 主题：（老年人 OR 老年 OR 老人） AND 主题：（共病 OR 多病共存 OR 多重慢性病 OR 慢性病共病 OR 多种慢性疾病 OR 共存疾病 OR 慢性病多病共存 OR 慢病共病） AND 主题：（患病率 OR 发病率 OR 患病情况）AND主题：(影响因素 OR 相关因素 OR 保护因素 OR 风险因素 OR 危险因素) AND 主题：(横断面研究 OR 横断面调查 OR 现况调查 OR 流行病学研究 OR 流行病学调查) | **111** |
| CBM |  | ("危险因素"[常用字段:智能] OR "保护因素"[常用字段:智能] OR "影响因素"[常用字段:智能] OR "相关因素"[常用字段:智能]) AND ("老年人"[常用字段:智能] OR "老年"[常用字段:智能]) AND ("共病"[常用字段:智能] OR "多病共存"[常用字段:智能] OR "共存疾病"[常用字段:智能] OR "多种慢性疾病"[常用字段:智能] OR "慢性病共病"[常用字段:智能]) AND ("横断面研究"[常用字段:智能] OR "横断面调查"[常用字段:智能] OR "流行病学调查"[常用字段:智能] OR "流行病学研究"[常用字段:智能]) | **106** |
| VIP |  | M=（共病 OR 多病共存 OR 多重慢性病 OR 慢性病共病 OR 多种慢性疾病 OR 共存疾病 OR 慢性病多病共存 OR 慢病共病) AND M=（患病率 OR 发病率 OR 患病情况 OR 影响因素 OR 危险因素 OR 保护因素 OR 相关因素） AND M=（老年人 OR 老年）AND M=(横断面研究 OR 横断面调查 OR 现况调查 OR 流行病学研究 OR 流行病学调查) | **198** |

Supplementary Table 3. Methodological quality assessment for included studies.

| **Studies included** | **①** | **②** | **③** | **④** | **⑤** | **⑥** | **⑦** | **⑧** | **⑨** | **⑩** | **⑪** | **Total score** | **Quality grade** |
| --- | --- | --- | --- | --- | --- | --- | --- | --- | --- | --- | --- | --- | --- |
| Liu YT et al.2022 | 1 | 1 | 1 | 1 | 0 | 1 | 1 | 1 | 0 | 1 | 0 | 8 | High |
| Chen YT et al.2023 | 1 | 1 | 1 | 1 | 0 | 1 | 1 | 1 | 0 | 1 | 0 | 8 | High |
| Li GX et al.2023 | 1 | 1 | 1 | 1 | 0 | 1 | 1 | 1 | 0 | 1 | 0 | 8 | High |
| Guo D et al.2022 | 1 | 1 | 1 | 1 | 0 | 1 | 0 | 1 | 0 | 1 | 0 | 7 | Medium |
| Kong Y et al.2021 | 1 | 0 | 1 | 1 | 0 | 1 | 0 | 0 | 0 | 0 | 0 | 4 | Medium |
| Li X et al.2019 | 1 | 1 | 1 | 1 | 0 | 1 | 0 | 1 | 0 | 0 | 0 | 6 | Medium |
| Zhang H et al.2019 | 1 | 0 | 1 | 1 | 0 | 1 | 0 | 0 | 0 | 1 | 0 | 5 | Medium |
| Qi YT et al.2023 | 1 | 1 | 1 | 1 | 0 | 1 | 0 | 1 | 0 | 1 | 0 | 7 | Medium |
| Liu XX et al.2023 | 1 | 1 | 1 | 1 | 0 | 1 | 0 | 0 | 0 | 0 | 0 | 5 | Medium |
| Tian L et al.2023 | 1 | 0 | 1 | 1 | 0 | 1 | 0 | 0 | 0 | 1 | 0 | 5 | Medium |
| Zhu PY et al.2023 | 1 | 1 | 1 | 1 | 0 | 1 | 0 | 1 | 0 | 1 | 0 | 7 | Medium |
| Yu ZJ et al.2023 | 1 | 1 | 1 | 1 | 0 | 0 | 0 | 0 | 0 | 0 | 0 | 4 | Medium |
| He YZ et al.2023 | 1 | 1 | 1 | 1 | 0 | 1 | 1 | 1 | 0 | 1 | 0 | 8 | High |
| Wang WH et al.2024 | 1 | 1 | 1 | 1 | 0 | 1 | 0 | 0 | 0 | 0 | 0 | 5 | Medium |
| Mu YJ et al.2023 | 1 | 1 | 1 | 1 | 0 | 1 | 1 | 1 | 0 | 1 | 0 | 8 | High |
| Yao YL et al.2022 | 1 | 0 | 1 | 1 | 0 | 1 | 0 | 0 | 0 | 0 | 0 | 4 | Medium |
| Cao M et al.2021 | 1 | 1 | 1 | 1 | 0 | 1 | 1 | 1 | 0 | 1 | 0 | 8 | High |
| Hou YT et al.2020 | 1 | 1 | 1 | 1 | 0 | 1 | 1 | 1 | 0 | 0 | 0 | 6 | Medium |
| Liu DN et al.2023 | 1 | 1 | 1 | 1 | 0 | 1 | 0 | 0 | 0 | 0 | 0 | 5 | Medium |
| Zhang XQ et al.2024 | 1 | 1 | 1 | 1 | 0 | 1 | 1 | 1 | 0 | 1 | 0 | 8 | High |
| Zhou FK et al.2023 | 1 | 1 | 1 | 0 | 0 | 1 | 0 | 1 | 1 | 0 | 0 | 6 | Medium |
| Marzban M et al.2024 | 1 | 1 | 1 | 1 | 0 | 0 | 0 | 1 | 0 | 0 | 0 | 5 | Medium |
| Oliveira-Figueiredo DST et al.2024 | 1 | 0 | 1 | 1 | 0 | 1 | 0 | 1 | 1 | 1 | 0 | 7 | Medium |
| Ko S et al.2024 | 1 | 1 | 1 | 1 | 0 | 1 | 1 | 1 | 0 | 1 | 0 | 8 | High |
| Su W et al.2024 | 1 | 1 | 1 | 1 | 0 | 1 | 1 | 1 | 0 | 1 | 0 | 8 | High |
| Kohler S et al.2024 | 1 | 1 | 1 | 1 | 0 | 1 | 1 | 0 | 0 | 1 | 0 | 7 | Medium |
| Lee C et al.2024 | 1 | 0 | 1 | 1 | 0 | 1 | 0 | 1 | 0 | 1 | 0 | 6 | Medium |
| Maimaitiwusiman Z et al.2023 | 1 | 1 | 1 | 1 | 0 | 1 | 0 | 1 | 0 | 1 | 0 | 7 | Medium |
| Reyes-Ortiz CA et al.2023 | 1 | 1 | 1 | 1 | 0 | 1 | 1 | 1 | 0 | 0 | 0 | 7 | Medium |
| You L et al.2023 | 1 | 1 | 1 | 1 | 0 | 1 | 1 | 1 | 1 | 0 | 0 | 8 | High |
| Yang K et al.2023 | 1 | 1 | 1 | 1 | 0 | 1 | 1 | 1 | 0 | 1 | 0 | 8 | High |
| Honda Y et al.2022 | 1 | 0 | 1 | 1 | 0 | 1 | 0 | 1 | 0 | 0 | 0 | 5 | Medium |
| Lynch DH et al.2022 | 1 | 1 | 1 | 1 | 0 | 1 | 0 | 1 | 0 | 0 | 0 | 6 | Medium |
| Balakrishnan S et al.2022 | 1 | 1 | 1 | 1 | 0 | 1 | 1 | 1 | 0 | 1 | 0 | 8 | High |
| Keomma K et al.2022 | 1 | 1 | 1 | 1 | 0 | 1 | 0 | 1 | 0 | 1 | 0 | 7 | Medium |
| Shariff Ghazali S et al.2021 | 1 | 0 | 1 | 1 | 0 | 1 | 0 | 1 | 0 | 0 | 0 | 5 | Medium |
| Lin WQ et al.2022 | 1 | 1 | 0 | 1 | 0 | 0 | 1 | 0 | 0 | 1 | 0 | 5 | Medium |
| Sara HH et al.2018 | 1 | 1 | 1 | 1 | 0 | 1 | 0 | 1 | 0 | 1 | 0 | 7 | Medium |
| Smith L et al.2022 | 1 | 1 | 1 | 1 | 0 | 1 | 1 | 1 | 0 | 1 | 0 | 8 | High |
| Jovic D et al.2016 | 1 | 0 | 1 | 1 | 0 | 1 | 0 | 1 | 0 | 0 | 0 | 5 | Medium |
| Puth MT et al.2017 | 1 | 0 | 1 | 1 | 0 | 1 | 0 | 0 | 0 | 1 | 0 | 5 | Medium |
| Bähler C et al.2015 | 1 | 1 | 1 | 1 | 0 | 1 | 0 | 0 | 0 | 0 | 0 | 5 | Medium |
| Ha NT et al.2015 | 1 | 0 | 1 | 1 | 0 | 1 | 1 | 1 | 0 | 1 | 0 | 7 | Medium |
| Yadav UN et al.2021 | 1 | 1 | 1 | 1 | 0 | 1 | 1 | 1 | 0 | 1 | 0 | 8 | High |
| Hien H et al.2014 | 1 | 1 | 1 | 1 | 0 | 1 | 1 | 1 | 0 | 1 | 0 | 8 | High |
| Aye SKK et al.2019 | 1 | 1 | 1 | 1 | 0 | 1 | 1 | 1 | 0 | 0 | 0 | 7 | Medium |
| Asante D et al.2022 | 1 | 1 | 1 | 1 | 0 | 1 | 0 | 1 | 0 | 1 | 0 | 7 | Medium |
| Abdulazeez ZU et al.2021 | 1 | 1 | 1 | 1 | 0 | 1 | 1 | 1 | 0 | 1 | 0 | 8 | High |
| Nugraha S et al.2020 | 1 | 0 | 1 | 1 | 0 | 1 | 1 | 0 | 0 | 0 | 0 | 5 | Medium |

Note:1.① Define the source of information (survey, record review); ② List inclusion and exclusion criteria for exposed and unexposed subjects (cases and controls) or refer to previous publications; ③ Indicate time period used for identifying patients; ④ Indicate whether or not subjects were consecutive if not population-based; ⑤ Indicated if evaluators of subjective components of study were masked to other aspects of status of the participants; ⑥ Describe any assessments undertaken for quality assurance purposes (e.g., test/retest of primary out come measurements); ⑦ Explain any patients exclusions from analysis; ⑧ Describe how confounding was assessed and/or controlled; ⑨ If applicable, explain how missing data were handled in the analysis; ⑩ Summarize patient response rates and completeness of data collection;⑪ If follow-up is available, identify the expected percentage of patients with incomplete data or follow-up outcomes.

2.“Yes” is assigned to 1, and “No or Unclear” is assigned to 0.
